# Comparing the effectiveness of pterostilbene and sitagliptin on modulating inflammatory levels and inducing autophagy to improve atherosclerosis outcome: A preclinical study in rabbits

**DOI:** 10.12688/f1000research.130682.2

**Published:** 2023-09-20

**Authors:** Hussam H Sahib, Bassim I Mohammad, Najah R Hadi, Azhar Al-Shaibany, Anil K Philip, Wisam J Mohammed, Dina A Jamil, Hayder A Al-Aubaidy

**Affiliations:** 1 Department of Pharmacology and Therapeutics, College of Pharmacy, University of Al-Qadisiyah, Al-Qadisiyah, Iraq; 2 Department of Pharmacology and Therapeutics, College of Medicine, University of Al-Qadisiyah, Al-Qadisiyah, Iraq; 3 Department of Pharmacology and Therapeutics, College of Medicine, University of Kufa, Al-Najaf, Iraq; 4 Health Service Executive, GIM Navan, Ireland; 5 School of Pharmacy, University of Nizwa, Birkat AlMouz, Oman; 6 Baghdad Medical Complex-Iraqi, Cardiology Center, Baghdad, Iraq; 7 Oceania University of Medicine, Melbourne, Australia; 8 Department of Microbiology, Anatomy, Physiology and Pharmacology & Centre for Cardiovascular Biology and Disease Research, School of Agriculture, Biomedicine & Environment, La Trobe University, Melbourne, VIC, 3086, Australia

**Keywords:** Pterostilbene, Sitagliptin, Atherosclerosis, PI3K, AMPK, AKT, Rabbits.

## Abstract

**Background**: Inflammation is the key contributor to the development of atherosclerotic plague. This study aims to evaluate the protective and autophagy induction properties of pterostilbene and sitagliptin on modulating the degree of atherosclerosis in rabbit models treated with an atherogenic diet.

**Methods**: 80 rabbits were randomly placed into one of four study groups (20 in each group): normal control diet (NC) fed normal diet for eight weeks, atherogenic control (AC) fed atherogenic diet for eight weeks, pterostilbene treated group (PT) fed atherogenic diet with pterostilbene (at 10 mg/kg/day) orally daily for eight weeks, and sitagliptin treated group (ST) fed atherogenic diet with sitagliptin (at 12 mg/kg/day) orally daily for eight weeks.

**Results**: While serum lipids and F2-isoprostane were elevated significantly in the AC study cohort compared to NC study cohort, ( *P* < 0.001), both pterostilbene and sitagliptin supplementations provided significant improvements in serum lipid parameters and F2-isoprostane in the PT study cohort and ST study cohort, respectively, when compared to the AC study cohort, ( *P*<0.001). Total cholesterol, triglycerides and LDL levels were significantly reduced among the PT and ST study cohorts as compared to the AC study cohort. This was coupled with a significant rise in LC3B levels (marker of tissue autophagy) among the PT study cohort and the ST study cohort, as compared to the AC study cohort, ( *P* < 0.001). The RNA expression of mTORC1 was reduced significantly at both PT study cohort and ST study cohort, ( *P*<0.001). Pterostilbene supplementation induced a significant reduction in tissue expression of PI3K and AKT, ( *P*<0.01), while sitagliptin induced significant reduction in 5’ adenosine monophosphate-activated protein kinase (AMPK) levels, ( *P*<0.001).

**Conclusions**: The results indicate that pterostilbene and/or sitagliptin supplementation can significantly improve the outcome of atherosclerosis due to their effects on the inflammatory pathways which hinder the progression of atherosclerotic plaque formation.

## Introduction

Atherosclerosis is a chronic inflammatory condition which affects various tissues and organs and can lead to serious complications including the development of cardiovascular disease, stroke, and diabetes mellites. ^1^ As part of disease progression, the atherosclerotic plaque could be detached from the vascular wall and can lead to a major cardiovascular complication. ^1^ There is still a knowledge gap about the exact mechanisms for plaque rupture, however, previous studies have illustrated the action of inflammation and oxidative damage in atherogenic plaques progression. ^2^ Hence, there is a close correlation among the degree of oxidation and inflammation and atherosclerosis, and so it is important to monitor the inflammatory markers as they may help to provide information about atherosclerosis progression which is of clinical usefulness.

Autophagy is a normal physiological mechanism through which the body can recycle cytoplasmic components (such as damaged/aged organelles and other cellular proteins), which are phagocytosed, and flagged for destruction by lysosomes. ^3^ Autophagy plays a key role in cellular homeostasis and can help get rid of damaged cells in disease conditions such as cancer and chronic illnesses. ^4^ Therefore, induction of autophagy in blood vessels can protect endothelial cells and smooth muscle cells damage, which may occur due to atherosclerosis and reduce the vulnerability of the plaques. ^4^ Macrophage autophagy can be useful in inhibiting atherosclerotic plaque rupture, which could reduce the severity of the condition. ^4^ mTOR is Ser/Thr protein kinase, considered to be a key control in cellular nutrition and energy expenditure, thus playing a central role in regulation of autophagy. ^5^ It can integrate multiple signals from upstream pathways and block the formation of autophagosomes. ^5^ Accordingly, there are several signaling paths which monitor autophagy induction. These include PI3K-Akt-mTOR and AMPK-mTOR pathways. ^6^ Previously, it was indicated that the (PI3K-Akt-mTOR) represent the primary pathway which is responsible for regulating a variety of cellular behaviors such as growth, apoptosis, and autophagy. ^6^ The activation of (PI3K/Akt/mTOR) signaling pathway in atherosclerosis may provide insight on the therapeutic role of inhibiting this pathway to control the development of atherosclerosis. LC3B is defined as an RNA-binding protein, which can be used to initiate mRNA degradation during autophagy, and hence will be measured in the current study to indicate the degree of autophagy. ^4^

Pterostilbene is a di-methylated analog of resveratrol. It has been known for its potent anti-inflammatory properties ^7^; hence it can be used to reduce the degree of inflammation and improve the outcome of atherosclerosis. It was previously shown that pterostilbene downregulates NF-κB and Toll like receptor 5 expression. ^8^ In addition, pterostilbene can inhibit smooth muscle activation via its effects on adenosine-monophosphate-activated-protein-kinase (AMPK) pathway. ^7^ This signaling pathway (AMPK - STAT3) provides an index about the degree of inflammation of the endothelial cells in the vascular wall. ^7^ Sitagliptin is a known oral antidiabetic medication of gliptins family, which is used as a second line treatment to treat hyperglycemia in patients with diabetes mellitus. ^9^ Gliptins work through their effects of stimulating insulin production and secretion via inhibiting dipeptidyl peptidase-4 enzyme and thus increasing the half-life of incretin hormones in response to diet. ^10^ Thus, the use of sitagliptin can help in improving insulin sensitivity and reduce the development of diabetic complications including atherosclerosis.

The current study aims to highlight the benefits of pterostilbene and sitagliptin supplementations in improving the outcome of atherosclerosis in rabbits treated with an atherogenic diet.

## Methods

### Ethical approval

All the procedures were performed according to the guidelines approved by the National Institutes of Health. The research study received ethics approval from the Animal Research Ethics, College of Medicine, University of Kufa, Iraq (approval no. 15792, on 14th December 2020) where the research took place. All experimental procedures involving animals were carried out in keeping with guidelines from the National Institutes of Health Guide for the Care and Use of Laboratory Animals to ameliorate any suffering of animals.

### Animal protocol

This study included 80 rabbits (New Zealand White; 26 males and 54 females). Rabbits were aged between 1-3 years old and weighed between 1300 grams – 3000 grams. All the rabbits were housed individually in polycarbonate cages (0.90 × 0.60 × 0.60 m) for two weeks on a 12-h light/dark cycle at a steady temperature of 25°C and humidity of 50% for acclimatization to the environment. Animals were fed a standard pellet diet with tap water ad libitum and were routinely inspected for food consumption and fecal characteristics. These animals have not been included in previous research. Animals were excluded if they appeared unwell prior to being included in the study.

### Study design

Following the first two weeks, animals were randomly divided into one of the four study groups (20 animals in each study group). A sample size of 20 animals in each group would present more than 85% power to detect significant differences with 0.45 effect size and at a significance level of α=0.05. Sample groups were chosen utilizing simple random sampling. Each animal was assigned a tag number, the blind-folded researcher (B.M.) then picked numbered tags from the hat.

• **Normal control study cohort (NC)**: In this group, rabbits received traditional chow diet and water ad libitum for eight weeks.
• **Atherogenic control study cohort (AC)**: In this group, animals were given an atherogenic diet, consisting of 2% cholesterol enhanced diet and water for 8 weeks.
• **Pterostilbene treated study cohort (PT)**: In this group, rabbits received an atherogenic diet, water and pterostilbene (purity 98%, Hangzhou Hyper Chemicals Limited, China; CAS No. 537-42-8) supplements at 10 mg/kg orally daily for 8 weeks.
• **Sitagliptin treated group (ST)**: In this group, rabbits received atherogenic diet, water and sitagliptin (Purity 98%, Hangzhou Hyper Chemicals Limited, China; Batch No. 20112301) supplements at 12 mg/kg orally daily for 8 weeks.

### Induction of atherosclerosis

To induce hyperlipidemia and subsequent development of atherosclerotic changes, animals were provided with 2% high cholesterol (BDH Chemicals Ltd, England), in their food to develop atherosclerotic changes in the aorta following 8 weeks supplementation. ^11^ During the study, animals were monitored on a daily basis to check their vital signs. In addition, blood pressure, body weight and blood samples were collected fortnightly to measure blood glucose levels.

At the conclusion of the study, rabbits were kept fasted overnight, then they were euthanized using ketamine (HIKMA Pharmaceuticals, 3310), using (66 mg/kg), and xylazine (Alfasan, 1004111-07), sing (6 mg/kg), via intramuscular injection. ^12^^,^^13^ Following euthanasia, thoracotomy was performed to expose the heart and collect blood. Aortic arch was dissected, and samples collected as well as the following:

• Serum lipid profile (total cholesterol – TC, triglyceride – TG, low density lipoprotein cholesterol – LDL, high density lipoprotein cholesterol – HDL, and very low-density lipoprotein cholesterol – VLDL. Serum lipids were measured using enzymatic methods (Abbott, Alcyon 300 Chemistry Analyzer, USA).
• Serum F2-isoprostane to assess lipid peroxidation. This has been assessed colormetrically via ELISA (Sunlong, China, SL0284Rb).
• Tissue LC3B as a marker of tissue autophagy marker using the LC3 Antibody Kit for Autophagy (Thermo Fisher, USA, catalogue number: L10382).
• Assessments of mTOR (PI3K, AKT, AMPK, and mTORC1) using RT-PCR (see details below).
• Histopathological examination of the aorta looking for atherosclerotic changes.

### Extraction of total RNA from aorta and reverse transcriptase polymerase chain reaction

Total RNA was extracted from heart tissue samples by using a TRIzol® reagent kit (Thermo Fisher, Catalogue number 12183555): 100 mg heart tissue sample was homogenized by adding 750 μl of TRIzol® reagent. Add 200 μl chloroform, and stir for 15 seconds, then place on ice for 5 minutes before centrifugation. Transfer 500 ul into a new tube and add 500 ul isopropanol and incubate in a fridge for 10 minutes, before centrifugation. Discard the supernatant, add 1 ml of ethanol and repeat the mixing and centrifugation as above. Discard the supernatant and leave the RNA pellet until dried out, then add 100 ul of free nuclease H _2_O to dissolve before RNA extraction.

As for the reverse transcriptase polymerase chain reaction (RT-PCR), using a mixture containing 3 μg total RNA, 2.5 μM oligo (dT), 1.5 mM magnesium chloride (MgCl _2_), 0.01 M dithiothreitol, and 200 units of SuperScript III reverse transcriptase in a total volume of 20 μl. The RT steps consisted of an RT step (42°C for 60 minutes) and a denaturation step (70°C for 5 minutes). The PCR steps consisted of a denaturation step (95°C for 30 seconds), a primer annealing step (60°C for 30 seconds), and an elongation step (72°C for 30 seconds) for 30 cycles. In the final step, the duration of the elongation step was seven minutes. Finally, the samples were loaded onto a 1% agarose gel. The gels were then observed and photographed under ultraviolet light.

### Statistical analysis

Means and standard error of means (SEM) were analyzed utilizing Statistical Package for Social Sciences (version 26, IBM, USA). One-way ANOVA for multiple comparisons followed by LSD-post-hoc test. Significant *P* value of <0.05 was considered. The Mann-Whitney test used to assess histopathological grading. A significant *P* value was set at ≤0.001.

## Results

### Atherogenic diet effects on lipid profile and atherogenic index

The mean serum levels of TC, TG, and LDL increased significantly in the AC study cohort versus the NC study cohort, ( *P*<0.001). Pterostilbene supplementation significantly reduced serum levels of TC, and LDL, while sitagliptin supplementation significantly reduced TC, TG, and LDL ( *P*<0.001) ( Table 1).

**Table 1.** Levels of serum lipid profile among the four study groups.

	Normal control group	Atherogenic control group	Pterostilbene treated group	Sitagliptin treated group
**TC (mg/dl)**	68.1±5.1	810.3±78.2	634.8±19.1 *	478.5±56.1 ^#^
**TG (mg/dl)**	47.8±1.5	294.5±25.6	201.3±16.7	150.1±19.4 ^#^
**LDL (mg/dl)**	25.3±2.8	647.3±70.3	347.1±20.9 *	274.6±31.8 ^#^
**HDL (mg/dl)**	15.6±0.8	24.6±0.5	21.3±0.7	18.7±0.9 ^#^
**F2-Isoprstane (pg/ml)**	167.9±7.5	798.3±43.1	523.4±25.8 *	598.3±45.9 ^#^

* Significance ( *P*<0.001) among the pterostilbene & the atherogenic study cohorts.

^#^ Significance ( *P*<0.01) among the sitagliptin & the atherogenic study cohorts.

TC: total cholesterol; TG: triglycerides; LDL: low density lipoprotein; HDL: high density lipoprotein.

Serum HDL levels were increased in the ST study cohort in comparison to the AC study cohort, ( *P*<0.01), something which was not achieved with the PT study cohort ( Table 1). In addition, mean serum HDL level was reduced significantly in the AC study cohort in comparison to the NC study cohort and then improved significantly following sitagliptin supplementation ( *P*<0.001) ( Table 1).

### Effect of atherogenic diet and treatment on oxidative stress maker F2-Isoprostane

**Figure 1.**
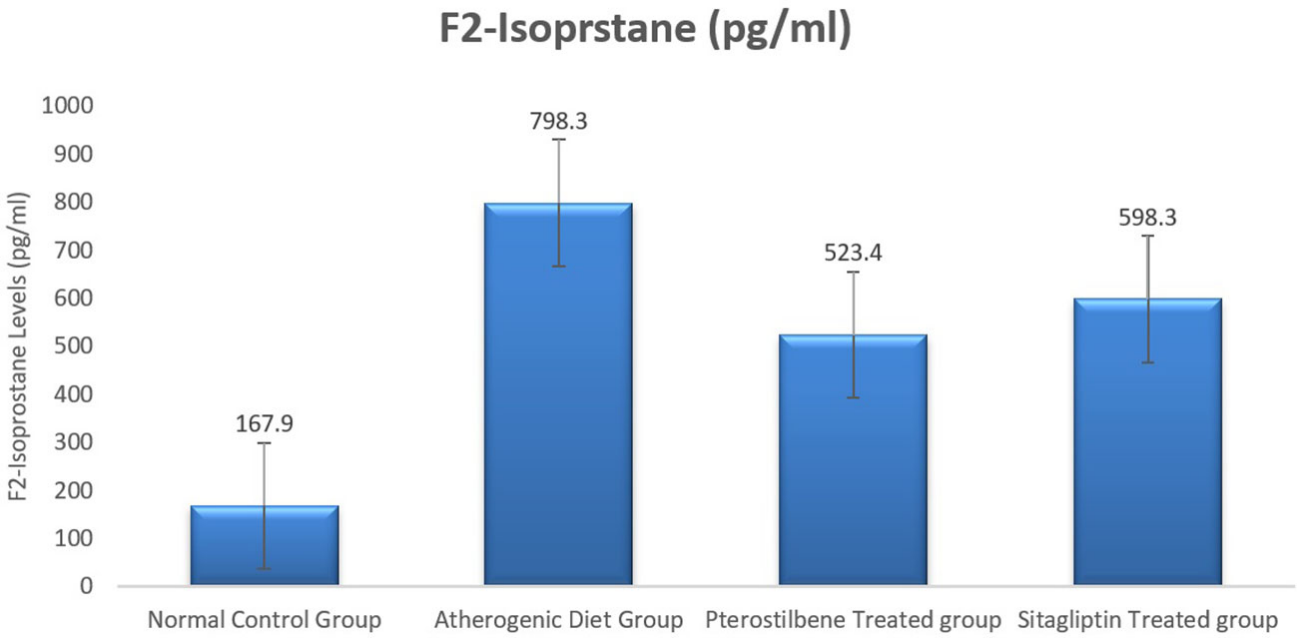
Plasma F2-Isoprostane levels among the four study groups ( *P*<0.005).

As a lipid peroxide marker, plasma F2-Isoprostane was significantly increased following the ingestion of atherogenic diet for 8 weeks (AC study cohort) when compared to the NC study cohort ( *P*<0.001). Moreover, the mean plasma level of F2-isoprostane was reduced significantly in response to 8 weeks supplementation of pterostilbene 523.4±72.4 pg/ml, and sitagliptin 598.3±59.3 pg/ml, when compared to the atherogenic study cohort 798.3±66.3 pg/ml ( *P*<0.05) ( Figure 1, Table 1).

### Effect of atherogenic diet and treatment options on aortic atherosclerotic lesion degree

**Figure 2.**
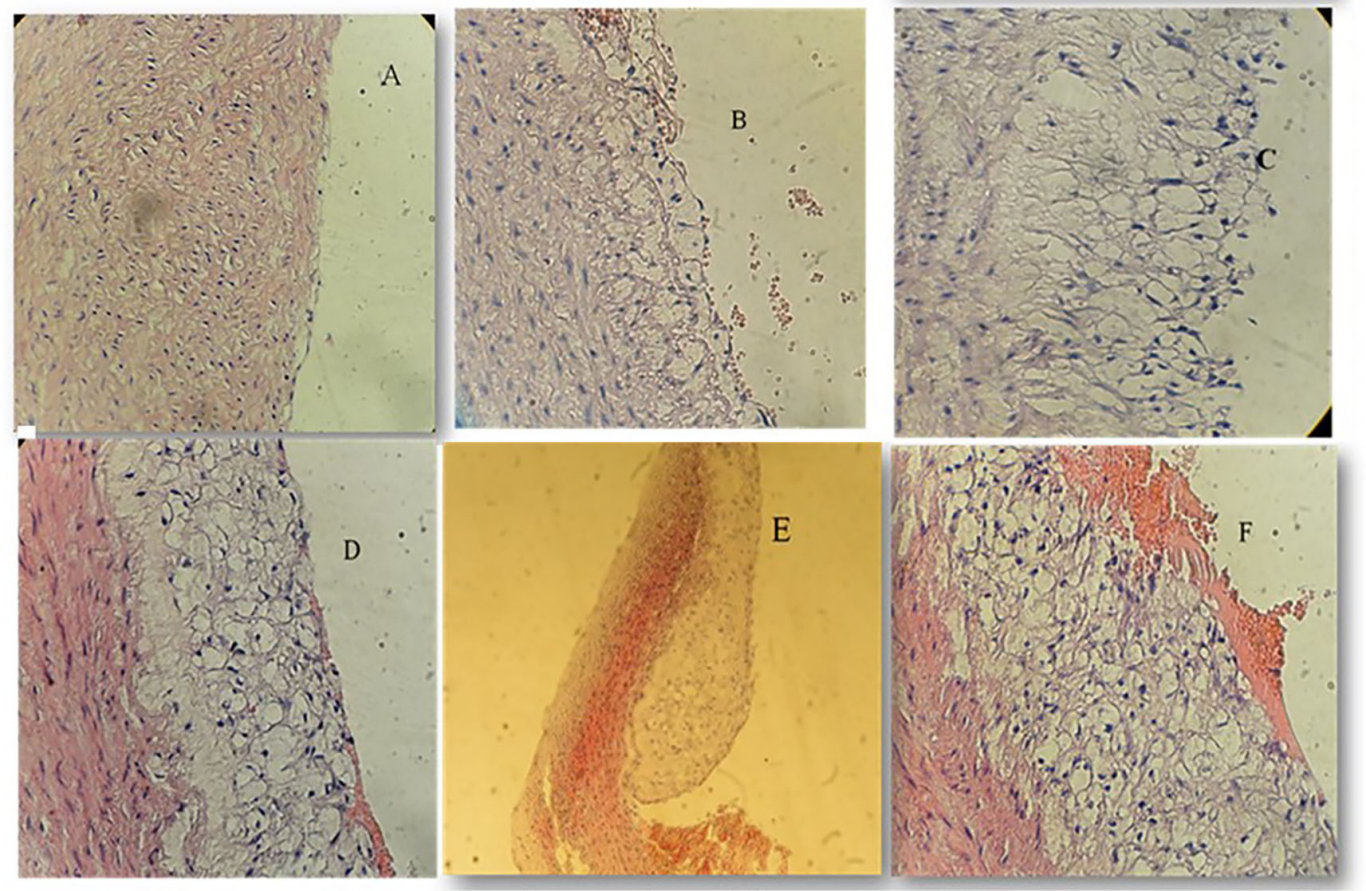
Atherosclerotic changes; cross-section in aortic arch; high fat diet rabbit; hematoxylin and eosin stain, (×40) for figures A, B, C, D, F, (x10) for Figure E. A: Normal arterial appearance in the control group; B: Initial atherosclerotic changes – lipid-laden foam cells in the sitagliptin study cohort; C: fatty streak and D: Intermediate atherosclerotic changes – extracellular lipid pool in the Pterostilbene study cohort; E: Advance atherosclerotic changes – a core of extracellular lipid and F: Complicated atherosclerotic changes – hemorrhagic thrombus as seen in the atherogenic study cohort (no supplementations).

Using the Mann-Whitney test to look for the significance across the four study groups, we identified significant reduction in the degree of atherosclerotic changes when using pterostilbene and or sitagliptin supplementation with atherogenic diet for 8 weeks ( *P*<0.001) ( Figure 2). Atherosclerotic changes were assessed following the American Heart Association classification of atherosclerosis: Type-1 (initial lesions), Type-2 (fatty streak lesions), Type-3 (moderate lesions), Type-4 (atheroma lesion), Type-5 (advance lesion), and Type-6 (complicated lesion).

We have used the scoring methodology to interpret the lesions ( Table 2).

**Table 2.** The difference in median atherosclerotic lesion degree between the 4 study groups.

	Normal diet control group N (%)	Atherogenic diet control group N (%)	Sitagliptin treated group N (%)	Pterostilbene treated group N (%)	*P* (Kruskal-Wallis)
**Atherosclerotic lesion degree**					<0.001
**Normal**	7 (100)	0 (0)	1 (14.3)	0 (0)	
**Initial**	0 (0)	0 (0)	4 (57.1)	5 (71.4)	
**Intermediate**	0 (0)	1 (14.3)	2 (28.6)	2 (28.6)	
**Advanced**	0 (0)	3 (42.9)	0 (0)	0 (0)	
**Complicated**	0 (0)	3 (42.9)	0 (0)	0 (0)	
**Total**	7 (100)	7 (100)	7 (100)	7 (100)	
**Median**	Normal	Advanced	Initial	Initial	
**Mean rank**	4.5	24.86	15	13.64	

**Figure 3.**
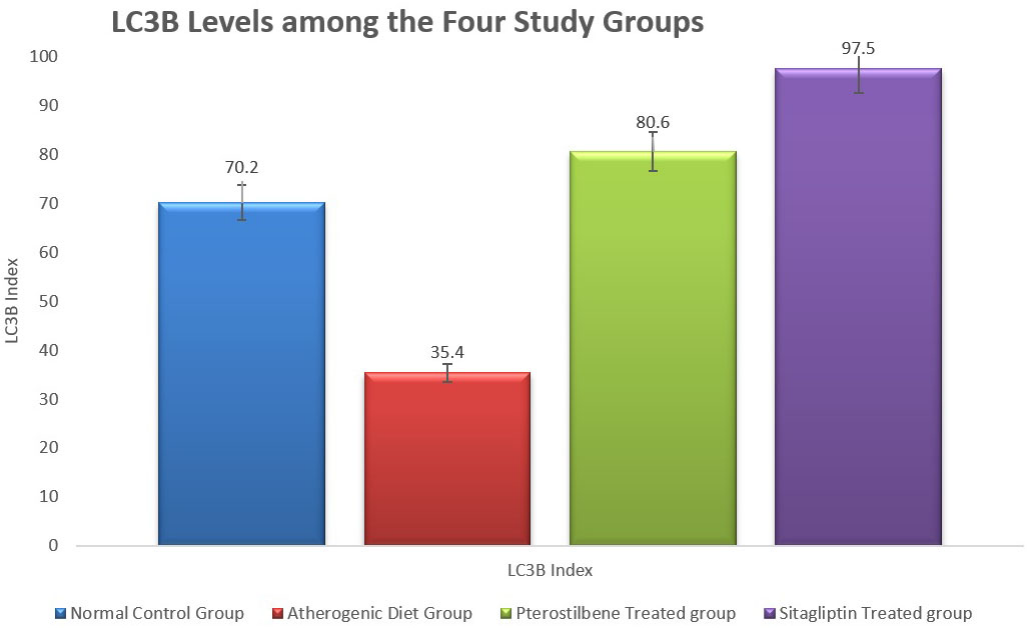
Marker of tissue autophagy (LC3B) in aortic tissue following the study conclusion ( *P*<0.001).

In addition, the tissue levels of LC3B were significantly reduced in the atherogenic control study cohort (35.4±7.8) compared to the NC study cohort (70.2±10.7, *P*<0.001). Both pterostilbene and sitagliptin supplementations appeared to induce significant improvements in the level of LC3B index, 80.6±14.3 and 97.5±21.2, respectively, in relation to the atherogenic study group ( Figure 3) ( *P*<0.001).

### Effect of pterostilbene and sitagliptin on mRNA expression levels of PI3K, AKT, AMPK and mTORC1 in aortic tissues

**Figure 4.**
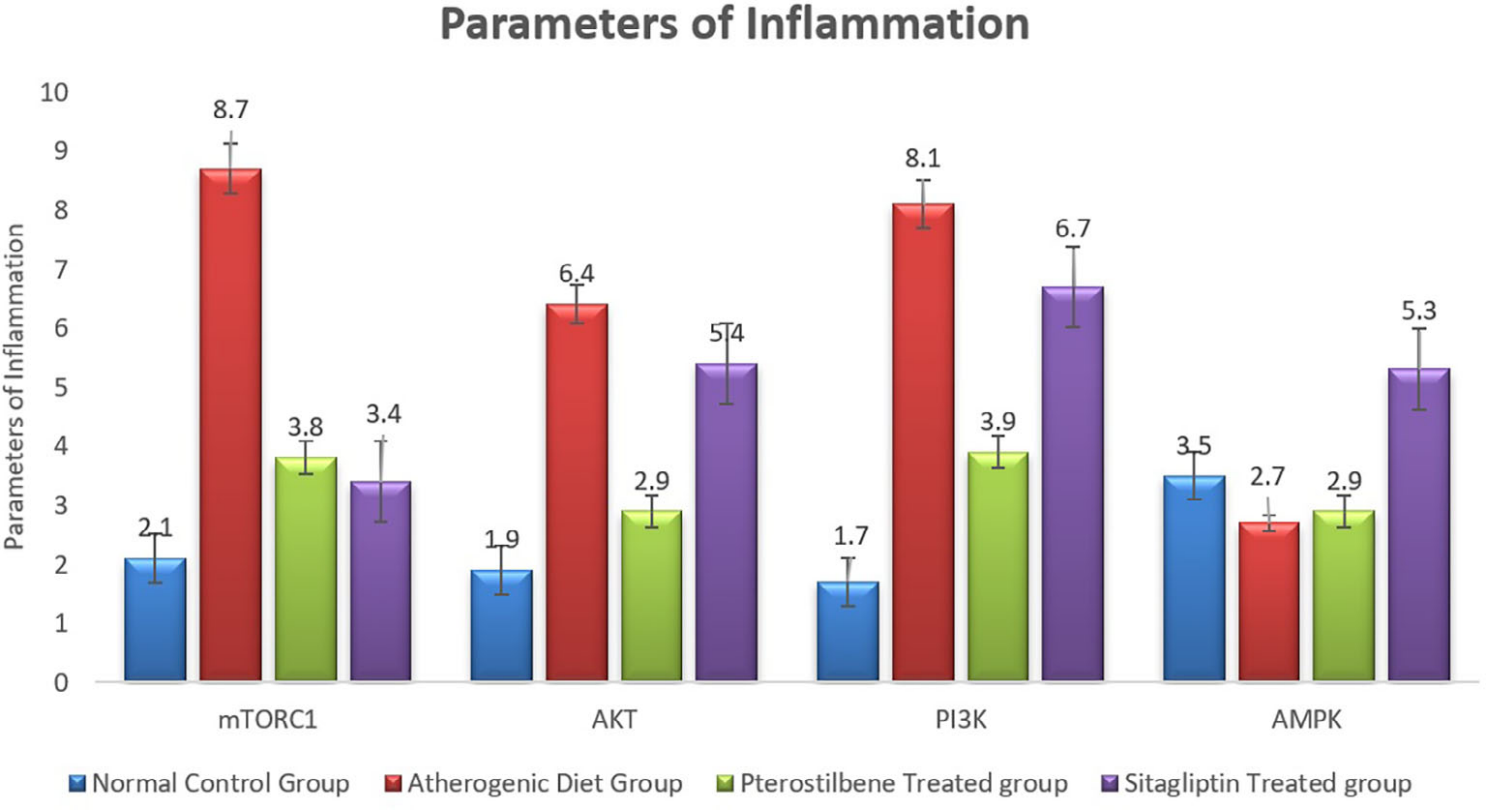
Markers of inflammatory changes (mTORC1, AKT, PI3K and AMPK) in aortic tissue following the end of the study ( *P*<0.001).

The atherogenic diet caused a significant increase in the degree of inflammation as manifested by the significant rise in the expression of mTORC1, PI3K and AKT ( *P*<0.001). Following the 8 weeks of supplementations, the expression of mTORC1 was significantly reduced in response to pterostilbene and sitagliptin supplementations as compared to the atherogenic study cohort ( *P*<0.001) ( Figure 4).

Pterostilbene supplementation exerted beneficial effect on improving the expression of aortic tissue levels of AKT and PI3K, something which could not be achieved with sitagliptin supplementation ( *P*<0.001) ( Figure 4).

Moreover, the mean AMPK index significantly dropped in the AC study cohort in comparison to the NC study cohort ( *P*<0.05). It then significantly increased following sitagliptin therapy ( *P*<0.001) ( Figure 4).

## Discussion

The current study compared the beneficial use of pterostilbene and sitagliptin supplementation on reducing the degree of inflammation and improving the outcome of atherosclerosis. We have demonstrated that the use of an atherogenic diet can significantly impair all components of lipids when compared to the normal control diet. ^14^ Following 8 weeks of supplementation in rabbits fed with atherogenic diet, both pterostilbene and sitagliptin significantly improved serum lipids in terms of significantly reducing TC and LDL and significantly increasing HDL levels. Previous studies revealed that pterostilbene induces similar effects to peroxisome proliferator-activated receptor α-isoform (PPAR-α) in reducing serum lipid profile. ^15^ In addition to its lipid lowering effects, PPAR-α can also reduce gene transcription for lipogenesis, a key step for lipid peroxidation which can precipitate the development of multiorgan disease, such as liver disease and heart failure. ^15^

We have also revealed a statistically significant reduction in plasma F2-Isoprostane in response to pterostilbene supplementation. Although we could not find any previous data related to this correlation, we hypothesize that this effect could be achieved due to the inhibitory effect of pterostilbene on the 8-iso-prostaglandin – alpha production. ^16^ F2-isoprostane level is a known marker for lipid peroxidation, and hence, it would make sense to measure this marker to get an estimation about the oxidized LDL and the degree of oxidative stress. ^17^

We have also identified increased mRNA expression of PI3K, AKT and mTORC1 in cholesterol-fed rabbits compared with normal control. ^18^ This can be explained due to the fact that oxidized LDL, a key risk factor for atherosclerosis development, can induce PI3K signaling in macrophages and foam cells, which can stimulate inflammatory cells’ synthesis and release which leads to monocyte chemotaxis, macrophage migration, and amplified intracellular lipid accumulation. The increased mTORC1 activity contributes to atherosclerosis progression in animal studies. ^19^ It is worth mentioning that earlier studies found that (PI3K-Akt-mTOR) pathway was associated with autophagy in vascular dysfunction. ^19^^,^^20^ As an example, the development of advanced glycation end products, due to persistent hyperglycemia, can inhibit the (PI3K-Akt-mTOR) pathway resulting in the development of serious vascular dysfunction. Hence, Pterostilbene can be used as an effective compound to inhibit the PI3K/Akt/mTOR pathway, regulates autophagy of macrophages and therefore may affect the progression of atherosclerotic plaque. ^19^^,^^20^ We have also revealed that LC3B level was significantly elevated following pterostilbene treatment, suggesting that the reduction in autophagosome formation in the atherogenic group was significantly prevented by pterostilbene treatment. ^21^

Finally, autophagy induction of macrophages may inhibit foam cell formation. This could be done via reducing oxidized LDL particles engulfing by macrophages and increasing cholesterol outflow into macrophages. These findings suggest that pterostilbene improves serum lipids, inhibits inflammation, and promotes autophagy which eventually improves the outcome of atherosclerotic plaque.

Moreover, this study illustrated that sitagliptin supplementation could increase expression of AMPK in aortic tissue when compared to the atherogenic group, something which could not be achieved with pterostilbene supplementation. Upregulation of AMPK following sitagliptin therapy could induce beneficial effects on atherosclerosis outcome by increasing plaque’s stability. ^22^ Sitagliptin supplementation was also beneficial in stimulating cellular autophagy via increasing tissue expression of LC3B ^23^ and suggests that the reduction in autophagosome formation in the atherogenic group was significantly prevented by sitagliptin treatment. Sitagliptin protects atherogenic rabbits against inflammation through reduction of macrophage accumulation, and prevention of the inflammatory pathway concurrent with improved autophagic processes via activation of the AMPK/mTORC1 pathway. ^23^

Due to the timeframe of this study, we could not measure the autophagy induction at other areas of the large blood vessels. In addition, scoring of atherosclerotic changes was calculated from the hematoxylin and eosin stain which may possess medium sensitivity. It would be best to use alternative, more sensitive measures for a higher accuracy using electron microscopy.

## Conclusion

We conclude that supplementation with pterostilbene or sitagliptin can significantly reduce the degree of oxidative stress, lipid peroxidation and inflammation which may ultimately help reduce the severity of atherosclerotic lesions in animal models with an atherogenic diet. This could be explained through the effects of these supplements on inhibiting inflammation through their effects on PI3K/Akt/mTOR, and AMPK/mTOR pathways.

## Author contributions

The authors responsibilities were as follows: Conceptualization, H.S., W.M., B.M., and N.H.; methodology, H.S., D.J., and N.H.; formal analysis, H.S., A.A., A.P., and H.A.; investigation, H.S., B.M., N.H., and H.A.; writing—original draft preparation, H.S., B.M., and N.H.; writing—review and editing, B.M., A.A., W.M., D.J., A.P., and H.A.; supervision, B.M., and N.H. All authors have read and agreed to the published version of the manuscript.

## Data Availability

figshare: Pterostilbene versus sitagliptin study, https://doi.org/10.6084/m9.figshare.22028744.v4. ^24^ This project contains the following data:
- Pterostilbene vs sitagliptin study. Data are available under the terms of the Creative Commons Attribution 4.0 International license (CC-BY 4.0). ARRIVE checklist for ‘Comparing the effectiveness of pterostilbene and sitagliptin on modulating inflammatory levels and inducing autophagy to improve atherosclerosis outcome: A preclinical study in rabbits’, https://doi.org/10.6084/m9.figshare.22028744.v4. ^24^
