# Comparing the effectiveness of pterostilbene and sitagliptin on modulating inflammatory levels and inducing autophagy to improve atherosclerosis outcome: A preclinical study in rabbits

**DOI:** 10.12688/f1000research.130682.4

**Published:** 2025-05-14

**Authors:** Hussam H Sahib, Bassim I Mohammad, Najah R Hadi, Azhar Al-Shaibany, Anil K Philip, Wisam J Mohammed, Dina A Jamil, Hayder A Al-Aubaidy

**Affiliations:** 1 Department of Pharmacology and Therapeutics, College of Pharmacy, University of Al-Qadisiyah, Al-Qadisiyah, Iraq; 2 Department of Pharmacology and Therapeutics, College of Medicine, University of Al-Qadisiyah, Al-Qadisiyah, Iraq; 3 Department of Pharmacology and Therapeutics, College of Medicine, University of Kufa, Al-Najaf, Iraq; 4 Health Service Executive, GIM Navan, Ireland; 5 School of Pharmacy, University of Nizwa, Birkat AlMouz, Oman; 6 Baghdad Medical Complex-Iraqi, Cardiology Center, Baghdad, Iraq; 7 Oceania University of Medicine, Melbourne, Australia; 8 Department of Microbiology, Anatomy, Physiology and Pharmacology & Centre for Cardiovascular Biology and Disease Research, School of Agriculture, Biomedicine & Environment, La Trobe University, Melbourne, VIC, 3086, Australia

**Keywords:** Pterostilbene, Sitagliptin, Atherosclerosis, PI3K, AMPK, AKT, Rabbits.

## Abstract

**Background:**

Inflammation is the key contributor to the development of atherosclerotic plague. This study aims to evaluate the protective and autophagy induction properties of pterostilbene and sitagliptin on modulating the degree of atherosclerosis in rabbit models treated with an atherogenic diet.

**Methods:**

80 rabbits were randomly placed into one of four study groups (20 in each group): normal control diet (NC) fed normal diet for eight weeks, atherogenic control (AC) fed atherogenic diet for eight weeks, pterostilbene treated group (PT) fed atherogenic diet with pterostilbene (at 10 mg/kg/day) orally daily for eight weeks, and sitagliptin treated group (ST) fed atherogenic diet with sitagliptin (at 12 mg/kg/day) orally daily for eight weeks.

**Results:**

While serum lipids and F2-isoprostane were elevated significantly in the AC study cohort compared to NC study cohort, ( *P* < 0.001), both pterostilbene and sitagliptin supplementations provided significant improvements in serum lipid parameters and F2-isoprostane in the PT study cohort and ST study cohort, respectively, when compared to the AC study cohort, ( *P*<0.001). Total cholesterol, triglycerides and LDL levels were significantly reduced among the PT and ST study cohorts as compared to the AC study cohort. This was coupled with a significant rise in LC3B levels (marker of tissue autophagy) among the PT study cohort and the ST study cohort, as compared to the AC study cohort, ( *P* < 0.001). The RNA expression of mTORC1 was reduced significantly at both PT study cohort and ST study cohort, ( *P*<0.001). Pterostilbene supplementation induced a significant reduction in tissue expression of PI3K and AKT, ( *P*<0.01), while sitagliptin induced significant increase in 5’ adenosine monophosphate-activated protein kinase (AMPK) levels, ( *P*<0.001).

**Conclusions:**

The results indicate that pterostilbene and/or sitagliptin supplementation can significantly improve the outcome of atherosclerosis due to their effects on the inflammatory pathways which hinder the progression of atherosclerotic plaque formation.

## Introduction

### Atherosclerosis and inflammation

Atherosclerosis is a chronic inflammatory condition that affects various tissues and organs, leading to serious complications such as cardiovascular disease, stroke, and diabetes mellites. ^1^ During disease progression, atherosclerotic plaques can detach from the vascular wall, resulting in major cardiovascular events. ^1^ While the exact mechanisms underlying plaque rupture remain unclear, several studies have highlighted the critical roles of inflammation and oxidative damage in the progression of atherogenic plaques. ^2^ For example, Libby et al. (2002) demonstrated that inflammatory cytokines, such as interleukin-6 (IL-6) and tumour necrosis factor-alpha (TNF-α), contribute to plaque instability and rupture by promoting matrix metalloproteinase (MMP) activity, which degrades the fibrous cap. ^2^ Additionally, Witold et al. (2017) identified oxidative stress as a key factor in endothelial dysfunction and foam cell formation, both of which accelerate atherogenesis. ^3^ These findings underscore the close correlation between the degree of oxidation, inflammation, and atherosclerosis progression. Therefore, monitoring inflammatory markers is clinically valuable, as they may provide critical insights into the progression of atherosclerosis.

### Role of autophagy in atherosclerosis

Autophagy is a normal physiological mechanism through which the body can recycle cytoplasmic components (such as damaged/aged organelles and other cellular proteins), which are phagocytosed, and flagged for destruction by lysosomes. ^4^ Autophagy plays a key role in cellular homeostasis and can help get rid of damaged cells in disease conditions such as cancer and chronic illnesses. ^5^ Therefore, induction of autophagy in blood vessels can protect endothelial cells and smooth muscle cells damage, which may occur due to atherosclerosis and reduce the vulnerability of the plaques. ^5^ Macrophage autophagy can be useful in inhibiting atherosclerotic plaque rupture, which could reduce the severity of the condition. ^5^ mTOR is Ser/Thr protein kinase, considered to be a key control in cellular nutrition and energy expenditure, thus playing a central role in regulation of autophagy. ^6^ It can integrate multiple signals from upstream pathways and block the formation of autophagosomes. ^6^ Accordingly, there are several signaling paths which monitor autophagy induction. These include PI3K-Akt-mTOR and AMPK-mTOR pathways. ^7^ Previously, it was indicated that the (PI3K-Akt-mTOR) represent the primary pathway which is responsible for regulating a variety of cellular behaviors such as growth, apoptosis, and autophagy. ^7^ The activation of (PI3K/Akt/mTOR) signaling pathway in atherosclerosis may provide insight on the therapeutic role of inhibiting this pathway to control the development of atherosclerosis. LC3B is defined as an RNA-binding protein, which can be used to initiate mRNA degradation during autophagy, and hence will be measured in the current study to indicate the degree of autophagy. ^5^

### Potential therapeutic agents: Pterostilbene and sitagliptin

Pterostilbene is a di-methylated analog of resveratrol, recognised for its potent anti-inflammatory properties, ^8^ making it a promising candidate for reducing inflammation and potentially improving atherosclerosis outcomes. Previous studies have demonstrated that pterostilbene downregulates NF-κB and Toll-like receptor 5 expression, ^9^ both of which are critical mediators of inflammatory responses in vascular diseases. Additionally, pterostilbene has been shown to inhibit smooth muscle activation through modulation of the adenosine-monophosphate-activated-protein-kinase (AMPK) pathway. ^7^ This AMPK-STAT3 signalling axis is a key indicator of endothelial inflammation within the vascular wall, suggesting a mechanistic pathway through which pterostilbene could exert protective effects against atherosclerosis. ^7^

Sitagliptin, an oral antidiabetic medication from the gliptin family, is commonly used as a second-line treatment to manage hyperglycaemia in patients with diabetes mellitus. ^10^ Gliptins function by stimulating insulin production and secretion through the inhibition of the dipeptidyl peptidase-4 enzyme, thereby prolonging the half-life of incretin hormones in response to dietary intake. ^11^ By improving insulin sensitivity, sitagliptin may also mitigate the development of diabetic complications, including atherosclerosis, given the close link between metabolic dysregulation and vascular inflammation.

### Study rationale and objectives

Despite extensive research on the individual roles of pterostilbene and sitagliptin, there remains a gap in understanding their combined effects on atherosclerosis, particularly in the context of diet-induced vascular inflammation. Most prior studies have focused on their isolated impacts within metabolic or inflammatory pathways without exploring potential synergistic effects. The current study aims to address this gap by investigating the benefits of pterostilbene and sitagliptin supplementation in improving atherosclerosis outcomes in rabbits fed an atherogenic diet. This research seeks to elucidate not only the individual contributions of these compounds but also their potential interactive mechanisms, providing new insights into combination therapies for atherosclerosis management.

## Methods

### Ethical approval

All the procedures were performed according to the guidelines approved by the National Institutes of Health. The research study received ethics approval from the Animal Research Ethics, College of Medicine, University of Kufa, Iraq (approval no. 15792, on 14th December 2020) where the research took place. All experimental procedures involving animals were carried out in keeping with guidelines from the National Institutes of Health Guide for the Care and Use of Laboratory Animals to ameliorate any suffering of animals.

### Animal protocol

This study included 80 New Zealand White rabbits (26 males and 54 females), aged between 1 and 3 years and weighing between 1300 and 3000 grams. All rabbits were housed individually in polycarbonate cages (0.90 × 0.60 × 0.60 m) for two weeks to allow for acclimatization to the environment. The housing conditions were maintained on a 12-hour light/dark cycle at a constant temperature of 25°C with 50% humidity.

During the acclimatization period, the rabbits were fed a standard pellet diet and had access to tap water ad libitum. Food consumption and faecal characteristics were routinely monitored to assess health status. Rabbits were excluded from the study if they appeared unwell before enrolment. None of the animals had been previously used in research.

Following acclimatization, the rabbits were assigned to experimental groups and fed an atherogenic diet composed of a traditional chow supplemented with 2% cholesterol to induce atherosclerosis. This diet was designed to promote the development of atherosclerotic lesions, providing a suitable model for evaluating the effects of the tested interventions. The diet’s composition was chosen based on established protocols to ensure consistency and reproducibility of atherosclerotic outcomes.

### Study design

Following a two-week acclimatization period, animals were randomly assigned to one of four study groups, with 20 animals per group. A sample size of 20 animals per group was determined through power analysis, providing more than 85% power to detect significant differences with an effect size of 0.45 at a significance level of α = 0.05. Randomisation was conducted using simple random sampling: each animal was assigned a tag number, and a blindfolded researcher (B.M.) randomly selected numbered tags from a hat to allocate animals to the groups.

### Study groups:

• **Normal Control (NC):** Rabbits received a traditional chow diet and water ad libitum for eight weeks.
• **Atherogenic Control (AC):** Rabbits were fed an atherogenic diet containing 2% cholesterol and provided water ad libitum for eight weeks.
• **Pterostilbene Treated (PT):** Rabbits received an atherogenic diet, water ad libitum, and pterostilbene supplements (purity 98%, Hangzhou Hyper Chemicals Limited, China; CAS No. 537-42-8) at a dose of 10 mg/kg orally daily for eight weeks.
• **Sitagliptin Treated (ST):** Rabbits received an atherogenic diet, water ad libitum, and sitagliptin supplements (purity 98%, Hangzhou Hyper Chemicals Limited, China; Batch No. 20112301) at a dose of 12 mg/kg orally daily for eight weeks.

### Justification for dosage and feeding duration

The selected dosages for pterostilbene (10 mg/kg) and sitagliptin (12 mg/kg) were based on previously published studies that demonstrated their efficacy in modulating inflammatory and metabolic pathways relevant to atherosclerosis without causing adverse effects. ^12^^,^^13^ The pterostilbene dosage was chosen considering its bioavailability and anti-inflammatory potential observed in preclinical studies, while the sitagliptin dose reflects effective glucose-lowering and vascular protective outcomes in animal models. ^12^ ^,^ ^13^

An eight-week feeding duration was chosen to ensure sufficient time for the development of diet-induced atherosclerotic changes and to allow for the therapeutic effects of the interventions to manifest. This duration aligns with established protocols in similar rabbit models of atherosclerosis, providing a balance between the progression of the disease and the ethical considerations of prolonged animal experimentation.

### Induction of atherosclerosis

To induce hyperlipidemia and subsequent development of atherosclerotic changes, animals were provided with 2% high cholesterol (BDH Chemicals Ltd, England), in their food to develop atherosclerotic changes in the aorta following 8 weeks supplementation. ^14^ During the study, animals were monitored on a daily basis to check their vital signs. In addition, blood pressure, body weight and blood samples were collected fortnightly to measure blood glucose levels.

At the conclusion of the study, rabbits were kept fasted overnight, then they were euthanized using ketamine (HIKMA Pharmaceuticals, 3310), using (66 mg/kg), and xylazine (Alfasan, 1004111-07), sing (6 mg/kg), via intramuscular injection. ^15^^,^^16^ Following euthanasia, thoracotomy was performed to expose the heart and collect blood. Aortic arch was dissected, and samples collected as well as the following:

• Serum lipid profile (total cholesterol – TC, triglyceride – TG, low density lipoprotein cholesterol – LDL, high density lipoprotein cholesterol – HDL, and very low-density lipoprotein cholesterol – VLDL. Serum lipids were measured using enzymatic methods (Abbott, Alcyon 300 Chemistry Analyzer, USA).
• Serum F2-isoprostane to assess lipid peroxidation. This has been assessed colormetrically via ELISA (Sunlong, China, SL0284Rb).
• Tissue LC3B as a marker of tissue autophagy marker using the LC3 Antibody Kit for Autophagy (Thermo Fisher, USA, catalogue number: L10382).
• Assessments of mTOR (PI3K, AKT, AMPK, and mTORC1) using RT-PCR (see details below).
• Histopathological examination of the aorta looking for atherosclerotic changes.

### Extraction of total RNA from aorta and reverse transcriptase polymerase chain reaction

#### RNA extraction

Total RNA was extracted from aorta tissue samples using the TRIzol ^®^ reagent kit (Thermo Fisher, Catalogue number 12183555). Approximately 100 mg of aorta tissue was homogenised in 750 μl of TRIzol ^®^ reagent. To this, 200 μl of chloroform was added, mixed vigorously for 15 seconds, and then placed on ice for 5 minutes. The mixture was centrifuged, and the aqueous phase (500 μl) was transferred to a new tube. An equal volume (500 μl) of isopropanol was added, and the sample was incubated at 4°C for 10 minutes before centrifugation. The supernatant was discarded, and the RNA pellet was washed with 1 ml of ethanol, followed by centrifugation. After discarding the supernatant, the RNA pellet was air-dried and resuspended in 100 μl of nuclease-free H _2_O for RNA extraction.

### RT-PCR analysis

For reverse transcriptase polymerase chain reaction (RT-PCR), a reaction mixture was prepared containing 3 μg of total RNA, 2.5 μM oligo (dT) primers, 1.5 mM magnesium chloride (MgCl _2_), 0.01 M dithiothreitol (DTT), and 200 units of SuperScript III reverse transcriptase in a total volume of 20 μl. The RT process included an initial reverse transcription step at 42°C for 60 minutes, followed by a denaturation step at 70°C for 5 minutes.

PCR amplification was carried out with the following thermal cycling conditions: initial denaturation at 95°C for 30 seconds, primer annealing at 60°C for 30 seconds, and elongation at 72°C for 30 seconds for 30 cycles. A final elongation step was performed at 72°C for 7 minutes.

### Genes of interest and normalisation

The specific genes analysed in this study include **PI3K**, **AKT**, **AMPK**, and **mTOR**, which are key regulators in the autophagy signalling pathway and are highly relevant to atherosclerosis. PI3K and AKT are involved in cell growth and survival pathways, AMPK regulates cellular energy homeostasis, and mTOR plays a central role in inhibiting autophagy. These genes were selected to provide insights into the molecular mechanisms underlying atherosclerotic progression and the therapeutic effects of the tested compounds.

Gene expression levels were normalised against the housekeeping gene **GAPDH**, selected for its stable expression across experimental conditions. Relative gene expression was quantified using the 2^(-ΔΔCt) method, ensuring accurate comparison of target gene expression levels across different samples.

### Measurement of Autophagy Marker (LC3B)

LC3B (microtubule-associated protein 1 light chain 3 beta) was chosen as a representative marker of autophagy due to its critical role in autophagosome formation. During autophagy, LC3B-I (cytosolic form) is lipidated to form LC3B-II, which associates with autophagosome membranes, serving as a reliable indicator of autophagic activity.

LC3B expression was quantified through RT-PCR using specific primers targeting LC3B mRNA. Additionally, the LC3B-II/LC3B-I ratio will be assessed to determine autophagy flux. The rationale for selecting LC3B as a marker is its well-established correlation with autophagic activity and its use as a standard autophagy biomarker in atherosclerosis-related studies.

### Histopathological examination

Aorta tissue samples were collected from all animals for histopathological examination. The tissues were fixed in 10% neutral buffered formalin, embedded in paraffin, and sectioned at 5 μm thickness. Sections were stained using Hematoxylin and Eosin (H&E) to evaluate general histological architecture.

### Scoring criteria

Histopathological grading of atherosclerotic lesions was performed based on a semi-quantitative scoring system evaluating the following parameters:

1. **Intimal Thickness:** Scored from 0 (normal) to 3 (severe thickening).
2. **Lipid Deposition:** Scored from 0 (none) to 3 (extensive lipid accumulation).
3. **Inflammatory Cell Infiltration:** Scored from 0 (no infiltration) to 3 (dense infiltration).
4. **Plaque Vulnerability Indicators:** Including necrotic core formation and fibrous cap thinning, scored from 0 (absent) to 3 (prominent).

Each slide was evaluated independently by two blinded pathologists to ensure consistency and reduce observer bias. The final score for each parameter was averaged between the two observers.

### Statistical analysis

The sample size of 80 rabbits (26 males and 54 females) was determined based on a power analysis conducted prior to the study, aiming to achieve a statistical power of 80% with an alpha level of 0.05. This calculation was performed using G*Power software, considering the expected effect size derived from preliminary data and previous studies on atherosclerosis interventions. The unequal distribution of males and females was due to availability constraints and to ensure representation of both sexes, allowing for potential assessment of sex-based differences in response to the interventions.

Means and standard error of the mean (SEM) were calculated and analysed using the Statistical Package for Social Sciences (SPSS, version 26, IBM, USA). For multiple group comparisons, one-way ANOVA was performed, followed by the Least Significant Difference (LSD) post-hoc test to identify specific group differences. A P-value of <0.05 was considered statistically significant.

For histopathological grading, the non-parametric **Kruskal-Wallis test** was used to compare histopathological scores among all four groups. When significant differences were identified, **pairwise comparisons** were conducted using the **Mann-Whitney U test** to assess differences between specific groups, including direct comparisons between the pterostilbene-treated (PT) and sitagliptin-treated (ST) groups. A P-value of ≤ 0.001 was considered statistically significant for these analyses to control for Type I errors due to multiple comparisons.

## Results

### Atherogenic diet effects on lipid profile and atherogenic index

The atherogenic diet significantly elevated serum levels of total cholesterol (TC), triglycerides (TG), and low-density lipoprotein (LDL) compared to the NC cohort (P < 0.001). Pterostilbene supplementation notably reduced TC and LDL levels, while sitagliptin supplementation resulted in significant reductions in TC, TG, and LDL levels (P < 0.001) ( Table 1).

**Table 1.** Levels of serum lipid profile among the four study groups.

	Normal control group	Atherogenic control group	Pterostilbene treated group	Sitagliptin treated group
**TC (mg/dl)**	68.1±5.1	810.3±78.2	634.8±19.1 *	478.5±56.1 ^#^
**TG (mg/dl)**	47.8±1.5	294.5±25.6	201.3±16.7	150.1±19.4 ^#^
**LDL (mg/dl)**	25.3±2.8	647.3±70.3	347.1±20.9 *	274.6±31.8 ^#^
**HDL (mg/dl)**	15.6±0.8	24.6±0.5	21.3±0.7	18.7±0.9 ^#^
**F2-Isoprstane (pg/ml)**	167.9±7.5	798.3±43.1	523.4±25.8 *	598.3±45.9 ^#^

* Significance ( *P*<0.001) among the pterostilbene & the atherogenic study cohorts.

^#^ Significance ( *P*<0.01) among the sitagliptin & the atherogenic study cohorts.

TC: total cholesterol; TG: triglycerides; LDL: low density lipoprotein; HDL: high density lipoprotein.

High-density lipoprotein (HDL) levels were reduced in the PT and ST groups compared to the AC group (P<0.01), indicating that neither treatment restored HDL to normal levels ( Table 1).

### Effect of atherogenic diet and treatment on oxidative stress maker F2-Isoprostane

Plasma F2-Isoprostane levels, a marker of lipid peroxidation, were significantly elevated in rabbits fed an atherogenic diet for 8 weeks (AC cohort) compared to the normal control group (NC cohort) (P < 0.001). Supplementation with pterostilbene and sitagliptin for 8 weeks resulted in a significant reduction in plasma F2-Isoprostane levels compared to the AC cohort (P < 0.05).

**Figure 1.**
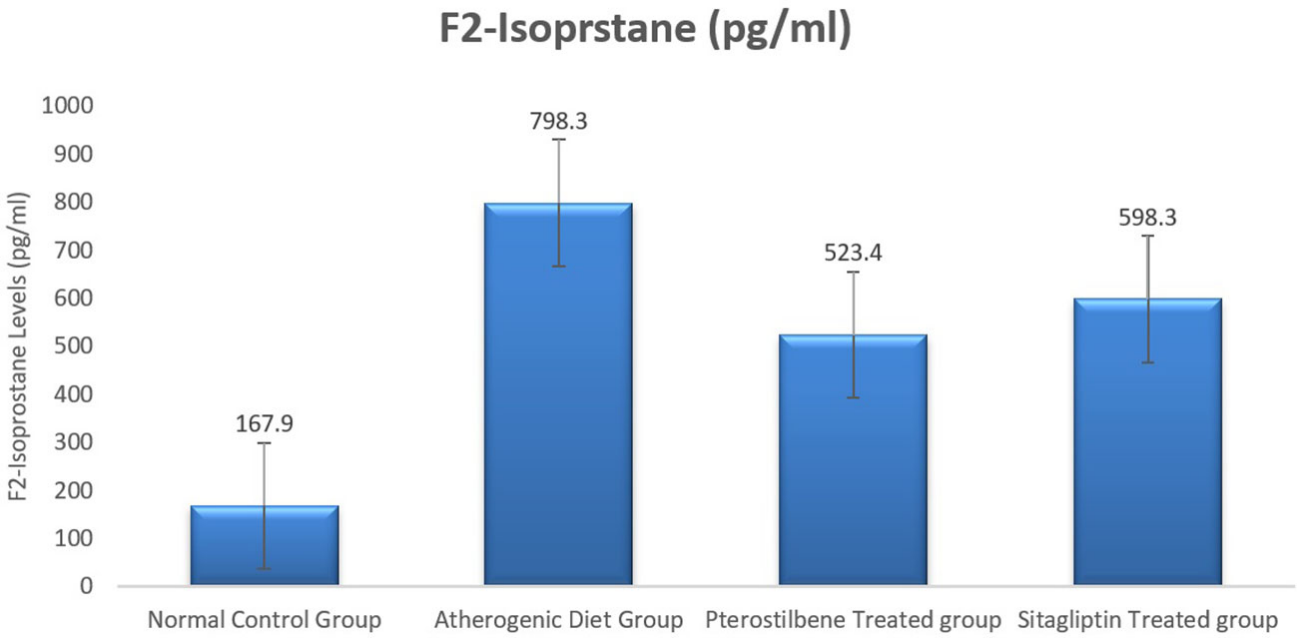
Plasma F2-Isoprostane levels among the four study groups ( *P*<0.005).

Key trends observed include a notable decrease in oxidative stress markers in response to both treatments, highlighting their potential antioxidative effects. Detailed numerical data are presented in Table 1, and the comparative trends are visually depicted in Figure 1, ensuring a clear representation without redundancy.

### Effect of atherogenic diet and treatment options on aortic atherosclerotic lesion degree

Histopathological analysis revealed pronounced progression of atherosclerotic lesions in the AC cohort, characterised by advanced lesions with extracellular lipid cores and complicated lesions marked by hemorrhagic thrombus formation. These changes were significantly more severe compared to the NC cohort, which exhibited normal arterial architecture (P < 0.001).

In contrast, the PT and ST treated groups demonstrated marked improvements in aortic histopathology. The majority of rabbits in these groups exhibited initial or intermediate lesions with significantly reduced lipid accumulation and less pronounced intimal thickening. Pterostilbene treatment was associated with improved vascular integrity, while sitagliptin treatment resulted in reduced foam cell formation and inflammatory infiltration.

**Figure 2.**
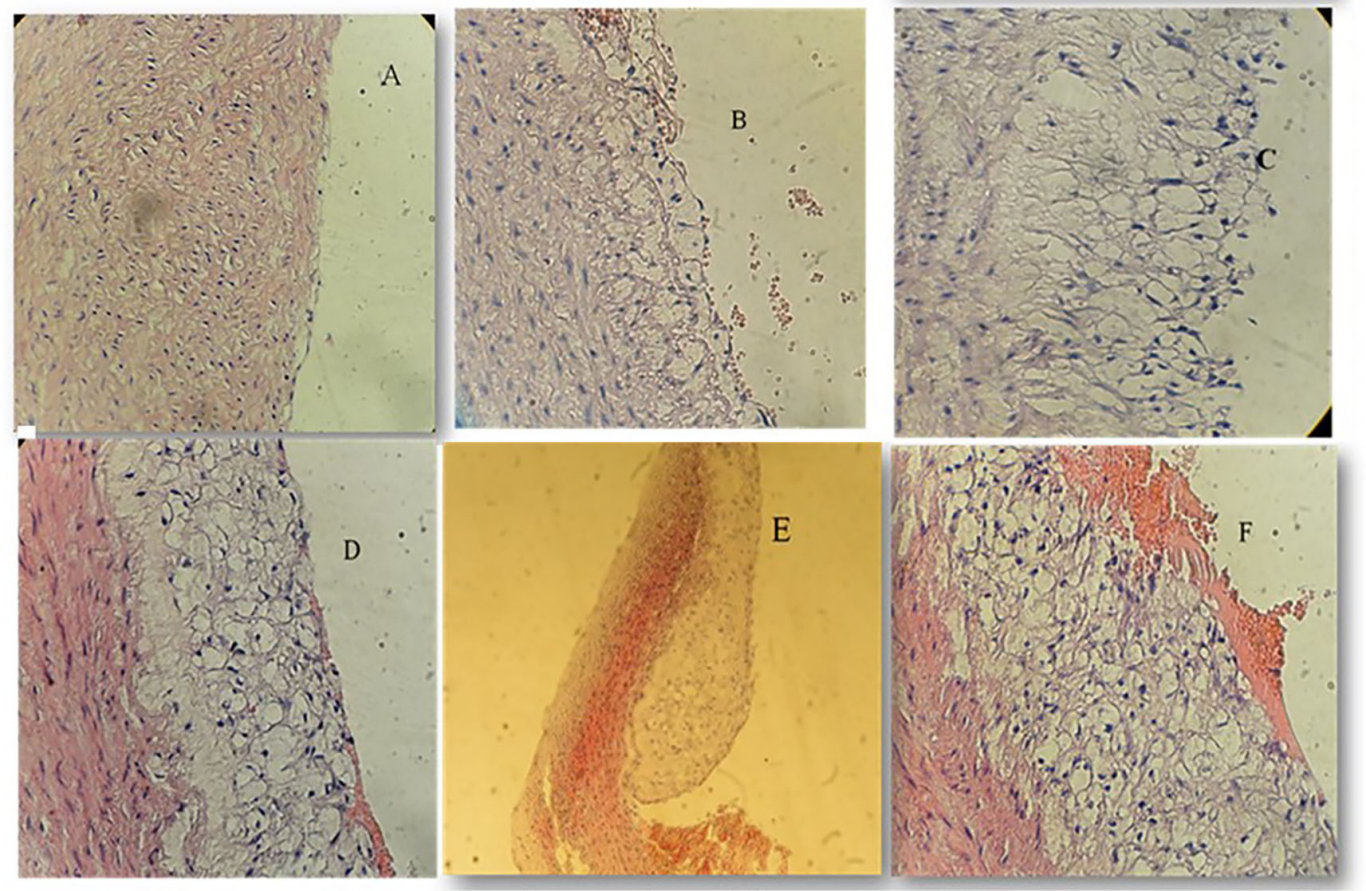
Atherosclerotic changes; cross-section in aortic arch; high fat diet rabbit; hematoxylin and eosin stain, (×40) for figures A, B, C, D, F, (x10) for Figure E. A: Normal arterial appearance in the control group; B: Initial atherosclerotic changes – lipid-laden foam cells in the sitagliptin study cohort; C: fatty streak and D: Intermediate atherosclerotic changes – extracellular lipid pool in the Pterostilbene study cohort; E: Advance atherosclerotic changes – a core of extracellular lipid and F: Complicated atherosclerotic changes – hemorrhagic thrombus as seen in the atherogenic study cohort (no supplementations).

The differences in lesion severity among the groups were statistically significant (P < 0.001, Kruskal-Wallis test), with pairwise comparisons confirming significant improvements in both PT and ST groups compared to the AC cohort. These findings are summarised in Table 2, with representative histological images provided in Figure 2 to illustrate the progression and regression of atherosclerotic lesions.

**Table 2.** The difference in median atherosclerotic lesion degree between the 4 study groups.

	Normal diet control group N (%)	Atherogenic diet control group N (%)	Sitagliptin treated group N (%)	Pterostilbene treated group N (%)	*P* (Kruskal-Wallis)
**Atherosclerotic lesion degree**					<0.001
**Normal**	7 (100)	0 (0)	1 (14.3)	0 (0)	
**Initial**	0 (0)	0 (0)	4 (57.1)	5 (71.4)	
**Intermediate**	0 (0)	1 (14.3)	2 (28.6)	2 (28.6)	
**Advanced**	0 (0)	3 (42.9)	0 (0)	0 (0)	
**Complicated**	0 (0)	3 (42.9)	0 (0)	0 (0)	
**Total**	7 (100)	7 (100)	7 (100)	7 (100)	
**Median**	Normal	Advanced	Initial	Initial	
**Mean rank**	4.5	24.86	15	13.64	

We have used the scoring methodology to interpret the lesions ( Table 2).

**Figure 3.**
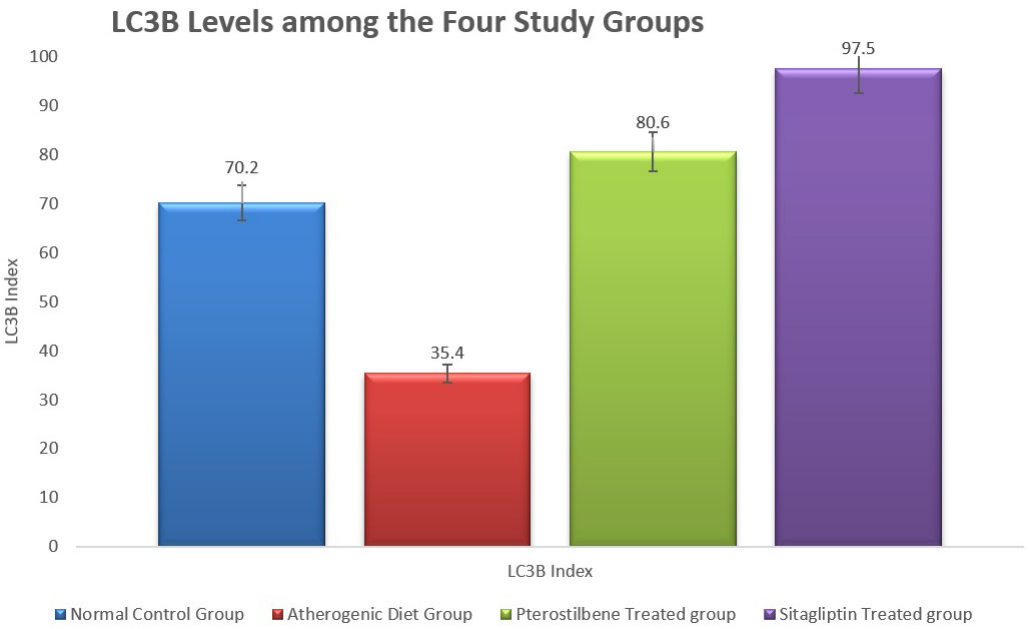
Marker of tissue autophagy (LC3B) in aortic tissue following the study conclusion ( *P*<0.001).

In addition, the Tissue levels of LC3B, a marker of autophagy, were significantly reduced in the AC cohort compared to the NC cohort (P < 0.001). Both pterostilbene and sitagliptin supplementation significantly increased LC3B levels compared to the AC cohort (P < 0.001), indicating enhanced autophagic activity ( Figure 3).

### Effect of pterostilbene and sitagliptin on mRNA expression levels of PI3K, AKT, AMPK and mTORC1 in aortic tissues

**Figure 4.**
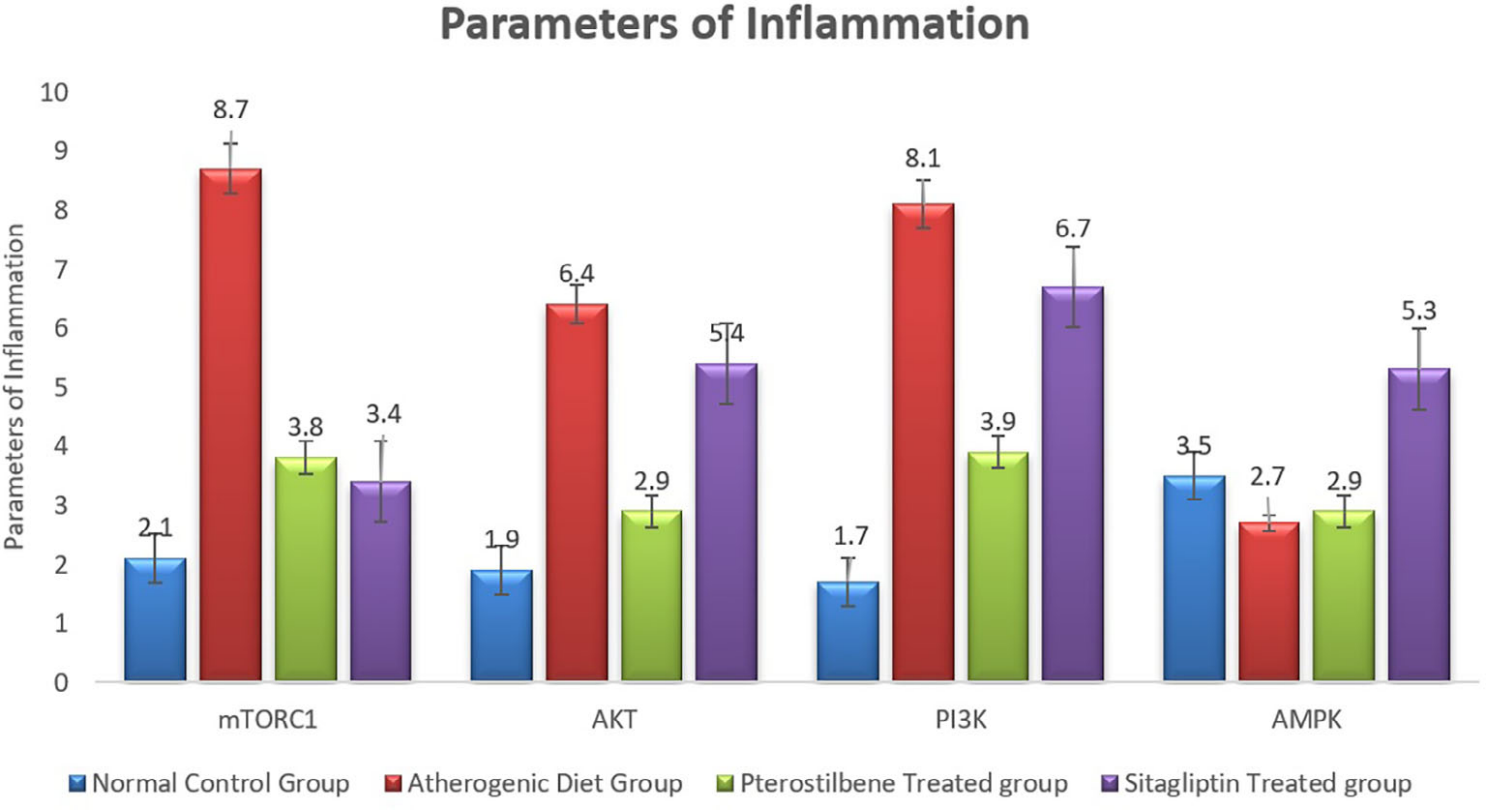
Markers of inflammatory changes (mTORC1, AKT, PI3K and AMPK) in aortic tissue following the end of the study ( *P*<0.001).

The atherogenic diet caused a significant increase in the degree of inflammation as manifested by the significant rise in the expression of mTORC1, PI3K and AKT (P < 0.001). Following the 8 weeks of supplementations, the expression of mTORC1 was significantly reduced in response to pterostilbene and sitagliptin supplementations as compared to the atherogenic study cohort (P < 0.001) ( Figure 4).

Pterostilbene supplementation significantly improved the expression of AKT and PI3K in aortic tissue compared to the AC cohort (P < 0.001), an effect not observed with sitagliptin treatment. Additionally, the AMPK index was significantly reduced in the AC cohort compared to the NC cohort (P < 0.05). Sitagliptin supplementation resulted in a significant increase in AMPK expression compared to the AC cohort (P < 0.001) ( Figure 4).

## Discussion

The current study evaluated the effects of pterostilbene and sitagliptin supplementation on reducing inflammation and improving atherosclerosis outcomes in a rabbit model. Our findings demonstrate that an atherogenic diet significantly impairs lipid profiles, as evidenced by increased total cholesterol (TC), triglycerides (TG), and low-density lipoprotein (LDL) levels, along with decreased high-density lipoprotein (HDL) levels compared to the normal control diet. ^17^

Following 8 weeks of supplementation, both pterostilbene and sitagliptin significantly improved total cholesterol, triglycerides, and LDL levels; however, HDL levels were lower in the treatment groups than in the atherogenic control group. This unexpected finding warrants further investigation, as HDL reductions may reflect treatment-specific effects on lipid metabolism or reverse cholesterol transport mechanisms. ^18^ Pterostilbene’s lipid-lowering effects may mimic those of peroxisome proliferator-activated receptor alpha (PPAR-α) agonists, known to reduce lipogenesis and lipid peroxidation, which are key drivers of multiorgan diseases such as liver disease and heart failure. ^18^ However, while these molecular mechanisms are plausible, they are based on indirect evidence and warrant further experimental validation.

Clinically, the observed improvements in lipid profiles are relevant, as dyslipidemia is a major risk factor for atherosclerosis. Compared to existing lipid-lowering therapies like statins, pterostilbene and sitagliptin may offer complementary benefits, particularly through their anti-inflammatory and autophagy-modulating effects. Future research should compare these agents directly with standard treatments to determine their relative efficacy and potential for clinical application.

Our study also revealed a significant reduction in plasma F2-isoprostane levels following pterostilbene supplementation, suggesting decreased oxidative stress. Although direct evidence linking pterostilbene to F2-isoprostane modulation is limited, we hypothesise that this effect may be mediated by the inhibition of 8-iso-prostaglandin-α production. ^19^ Since F2-isoprostanes are markers of lipid peroxidation, their reduction aligns with decreased oxidised LDL levels, further supporting the antioxidant potential of pterostilbene. ^20^

At the molecular level, we observed increased mRNA expression of PI3K, AKT, and mTORC1 in cholesterol-fed rabbits compared to controls, consistent with the role of oxidised LDL in activating PI3K signalling pathways. This activation promotes inflammatory responses, monocyte chemotaxis, and foam cell formation, contributing to atherosclerosis progression. ^21^ ^,^ ^22^ Pterostilbene appears to inhibit the PI3K/Akt/mTOR pathway, potentially regulating macrophage autophagy and influencing plaque stability. Elevated LC3B levels following pterostilbene treatment suggest enhanced autophagosome formation, indicating that pterostilbene may counteract the autophagy suppression observed in atherosclerotic conditions. ^23^ ^,^ ^24^

However, while autophagy induction can be protective, excessive autophagy may lead to cell death and exacerbate tissue damage. This dual role underscores the need for a balanced autophagic response, as both insufficient and excessive autophagy can contribute to atherosclerosis progression. Future studies should investigate the threshold at which autophagy shifts from being protective to detrimental in vascular tissues.

Sitagliptin supplementation was associated with increased AMPK expression in aortic tissue, an effect not observed with pterostilbene. AMPK activation is known to enhance plaque stability by promoting lipid metabolism, reducing inflammation, and improving endothelial function. ^25^ Beyond its glucose-lowering effects, sitagliptin may exert vascular protective actions through AMPK-mediated pathways, highlighting its potential utility in atherosclerosis management. Additionally, sitagliptin increased LC3B expression, suggesting enhanced autophagic activity, which may contribute to its anti-inflammatory effects and plaque-stabilising properties ^26^ and suggests that the reduction in autophagosome formation in the atherogenic group was significantly prevented by sitagliptin treatment. Sitagliptin protects atherogenic rabbits against inflammation through reduction of macrophage accumulation, and prevention of the inflammatory pathway concurrent with improved autophagic processes via activation of the AMPK/mTORC1 pathway.

Our study has several limitations. The assessment of autophagy was limited to the aorta, and additional vascular regions were not evaluated due to time constraints. Histopathological scoring relied on haematoxylin and eosin staining, which may lack the sensitivity of advanced imaging techniques such as electron microscopy. Moreover, the molecular mechanisms proposed are based on indirect evidence and require further validation through mechanistic studies.

Future research should focus on elucidating the precise molecular pathways involved in pterostilbene and sitagliptin’s effects on atherosclerosis. Clinical trials are needed to confirm their efficacy and safety in humans, with an emphasis on optimising dosing regimens and identifying potential synergistic effects with existing therapies. Investigating the long-term impact of these compounds on vascular health and their potential role in combination therapies could pave the way for novel strategies in atherosclerosis management.

## Conclusion

In conclusion, supplementation with pterostilbene or sitagliptin significantly reduced oxidative stress, lipid peroxidation, and inflammation in rabbits fed an atherogenic diet, which may ultimately help mitigate the severity of atherosclerotic lesions. These effects are potentially mediated through the modulation of inflammatory pathways, likely involving the PI3K/Akt/mTOR and AMPK/mTOR signalling cascades. However, it is important to interpret these findings with caution, as the mechanisms proposed are based on indirect evidence and require further experimental validation.

This study was conducted in an animal model, which may not fully replicate human pathophysiology. Therefore, while these results are promising, additional research, including well-designed human clinical trials, is necessary to confirm the efficacy and safety of pterostilbene and sitagliptin in managing atherosclerosis.

The potential clinical implications of these findings suggest that both pterostilbene and sitagliptin could serve as adjunctive therapies to existing atherosclerosis treatments. Furthermore, considering the distinct but complementary mechanisms of action observed, exploring combination therapy may offer synergistic benefits, enhancing therapeutic outcomes in atherosclerosis management. Future studies should investigate the optimal dosing strategies and the long-term effects of these compounds, both individually and in combination, to better understand their potential in clinical practice.

## Author contributions

The authors responsibilities were as follows: Conceptualization, H.S., W.M., B.M., and N.H.; methodology, H.S., D.J., and N.H.; formal analysis, H.S., A.A., A.P., and H.A.; investigation, H.S., B.M., N.H., and H.A.; writing—original draft preparation, H.S., B.M., and N.H.; writing—review and editing, B.M., A.A., W.M., D.J., A.P., and H.A.; supervision, B.M., and N.H. All authors have read and agreed to the published version of the manuscript.

## Data Availability

figshare: Pterostilbene versus sitagliptin study, https://doi.org/10.6084/m9.figshare.22028744.v4. ^27^ This project contains the following data:
- Pterostilbene vs sitagliptin study. Data are available under the terms of the Creative Commons Attribution 4.0 International license (CC-BY 4.0). ARRIVE checklist for ‘Comparing the effectiveness of pterostilbene and sitagliptin on modulating inflammatory levels and inducing autophagy to improve atherosclerosis outcome: A preclinical study in rabbits’, https://doi.org/10.6084/m9.figshare.22028744.v4. ^27^
