# Supplementary material for: Comparing the effectiveness of pterostilbene and sitagliptin on modulating inflammatory levels and inducing autophagy to improve atherosclerosis outcome: A preclinical study in rabbits
Source: F1000Res. 2025 May 14;12:339. Originally published 2023 Mar 27. [Version 4] doi: 10.12688/f1000research.130682.4 (PMC12223486; doi:10.12688/f1000research.130682.4)
Supplement: Peer review history

# Reviewer report: Reviewer response for version 4

**DOI:** 10.5256/f1000research.181862.r393700

**Published:** 2025-08-21

**Authors:** Marco Orecchioni

**Affiliations:** 1 Immunology Center of Georgia, Augusta University, Augusta, Georgia, USA

This revised manuscript by Sahib et al. highlights the role of pterostilbene and sitagliptin supplementation in mitigating the progression of atherosclerosis.

 Overall, the study is of interest; however, some points still require clarification.

1. The scope statement claims to investigate the combined effects of pterostilbene and sitagliptin, yet the data address only the individual compounds. To evaluate synergy, an additional group should be included in which rats receive both compounds simultaneously.
2. Control rats should also receive each compound while on a standard (non-atherogenic) diet to determine the supplements’ effects under steady-state conditions (especially on lipid content). This should be commented.
3. The authors did not examine the expression of key pro-inflammatory markers (e.g., TNF-α, IL-1β, IL-6, IL-10, TGF-β). Including these data would provide valuable mechanistic insight.
4. Because pterostilbene and sitagliptin affect cholesterol levels, rat body weights after treatment should be reported.
5. The observed reduction in atherosclerosis progression may stem from improved lipid profiles rather than a direct anti-inflammatory effect; this distinction should be discussed.
6. Are complete blood counts comparable across groups? Please provide these data or clarify.
7. A more thorough plaque analysis is warranted. For example, does supplementation alter macrophage content or plaque stability?
8. As a minor, the word "regression" should be used carefully since the data presented do not show regression, but rather a slower progression of the disease. Please adjust.

Is the work clearly and accurately presented and does it cite the current literature?

Yes

If applicable, is the statistical analysis and its interpretation appropriate?

Yes

Are all the source data underlying the results available to ensure full reproducibility?

Yes

Is the study design appropriate and is the work technically sound?

Partly

Are the conclusions drawn adequately supported by the results?

Partly

Are sufficient details of methods and analysis provided to allow replication by others?

Yes

Reviewer Expertise:

Macrophage biology, atherosclerosis, GPCRs, Olfactory receptors

I confirm that I have read this submission and believe that I have an appropriate level of expertise to confirm that it is of an acceptable scientific standard, however I have significant reservations, as outlined above.

# Reviewer report: Reviewer response for version 4

**DOI:** 10.5256/f1000research.181862.r384829

**Published:** 2025-07-02

**Authors:** Xin-Fang Leong

**Affiliations:** 1 Universiti Kebangsaan Malaysia, Bangi, Selangor, Malaysia

No further comments.  The manuscript is okay to be indexed.

Is the work clearly and accurately presented and does it cite the current literature?

Yes

If applicable, is the statistical analysis and its interpretation appropriate?

Yes

Are all the source data underlying the results available to ensure full reproducibility?

Yes

Is the study design appropriate and is the work technically sound?

Yes

Are the conclusions drawn adequately supported by the results?

Partly

Are sufficient details of methods and analysis provided to allow replication by others?

Partly

Reviewer Expertise:

Pharmacology, cardiovascular system, animal study

I confirm that I have read this submission and believe that I have an appropriate level of expertise to confirm that it is of an acceptable scientific standard.

# Reviewer report: Reviewer response for version 4

**DOI:** 10.5256/f1000research.181862.r384830

**Published:** 2025-05-28

**Authors:** Jelica Grujic Milanovic

**Affiliations:** 1 University of Belgrade, Belgrade, Serbia

I have no comment

Is the work clearly and accurately presented and does it cite the current literature?

Yes

If applicable, is the statistical analysis and its interpretation appropriate?

Yes

Are all the source data underlying the results available to ensure full reproducibility?

Yes

Is the study design appropriate and is the work technically sound?

Yes

Are the conclusions drawn adequately supported by the results?

Yes

Are sufficient details of methods and analysis provided to allow replication by others?

Yes

Reviewer Expertise:

polyphenols, resveratrol, oxidative stress, hyperlipidaemia, hypertension

I confirm that I have read this submission and believe that I have an appropriate level of expertise to confirm that it is of an acceptable scientific standard.

# Reviewer report: Reviewer response for version 3

**DOI:** 10.5256/f1000research.178031.r376390

**Published:** 2025-05-08

**Authors:** Xin-Fang Leong

**Affiliations:** 1 Universiti Kebangsaan Malaysia, Bangi, Selangor, Malaysia

The revised manuscript has shown marked improvement in terms of clarity, coherence, and overall quality. It is now well-written and considerably easier to understand. The authors have clearly addressed the previous comments, and the manuscript is much more compelling as a result. However, there are still some issues that need to be addressed before final acceptance.

 Firstly, there is a discrepancy (reduction vs. increase) between the mRNA expression findings for sitagliptin and pterostilbene reported in the abstract and those presented in the results section, particularly in the Figure 4. Please ensure these are consistent throughout the manuscript.

 Secondly, the statement regarding HDL levels requires clarification, as the current data indicate that HDL levels were increased in both the supplemented groups and the normal control group compared to the atherogenic diet group—this should be clearly reflected in the results/Table 1 and discussion.

 Thirdly, while H&E staining images have been provided, it would strengthen the histological analysis if Oil Red O staining images were also included, if available.

 Finally, the methodology section mentions that "PCR products were then separated on a 1% agarose gel, visualized, and photographed under ultraviolet light," but this data appears to be missing from both the manuscript and the supplementary materials. Please ensure that this data is either included or the statement is revised accordingly.

Is the work clearly and accurately presented and does it cite the current literature?

Yes

If applicable, is the statistical analysis and its interpretation appropriate?

Yes

Are all the source data underlying the results available to ensure full reproducibility?

Yes

Is the study design appropriate and is the work technically sound?

Yes

Are the conclusions drawn adequately supported by the results?

Partly

Are sufficient details of methods and analysis provided to allow replication by others?

Partly

Reviewer Expertise:

Pharmacology, cardiovascular system, animal study

I confirm that I have read this submission and believe that I have an appropriate level of expertise to confirm that it is of an acceptable scientific standard, however I have significant reservations, as outlined above.

# Response

**Published:** 2025-05-09

**Authors:** Hayder Al-Aubaidy

**Affiliations:** La Trobe University, Australia

Dear Dr Leong,

 Thank you very much for your review. Please see below our response:

**Reviewer (Dr. Xin-Fang Leong) Comments:**

 The revised manuscript has shown marked improvement in terms of clarity, coherence, and overall quality. It is now well-written and considerably easier to understand. The authors have clearly addressed the previous comments, and the manuscript is much more compelling as a result. However, there are still some issues that need to be addressed before final acceptance.

**Firstly**, there is a discrepancy (reduction vs. increase) between the mRNA expression findings for sitagliptin and pterostilbene reported in the abstract and those presented in the results section, particularly in the Figure 4. Please ensure these are consistent throughout the manuscript.

**Response:**

 We thank the reviewer for highlighting this inconsistency. We have carefully reviewed the data and confirmed that sitagliptin **increased** AMPK expression, as shown in Figure 4. Accordingly, we have revised the abstract to accurately reflect this finding. The sentence now reads:

 “Pterostilbene supplementation induced a significant reduction in tissue expression of PI3K and AKT, while sitagliptin induced a significant increase in 5’ adenosine monophosphate-activated protein kinase (AMPK) levels.”

 This correction ensures consistency across the abstract, results, and figures.

**Secondly**, the statement regarding HDL levels requires clarification, as the current data indicate that HDL levels were increased in both the supplemented groups and the normal control group compared to the atherogenic diet group—this should be clearly reflected in the results/Table 1 and discussion.

**Response:**

 We appreciate the reviewer’s careful examination of the lipid profile data. Upon reviewing Table 1, we confirm that **HDL levels were actually higher in the atherogenic control group than in the pterostilbene and sitagliptin groups**. To address this, we have revised the relevant sections as follows:

- In the **Results**, we state:

“HDL levels were significantly reduced in the PT and ST groups compared to the AC group (P<0.01), indicating that neither treatment restored HDL to normal levels.”

- In the **Discussion**, the paragraph has been updated to acknowledge the lower HDL levels in the treatment groups, and a note is added that this unexpected result may be due to compound-specific effects on lipid metabolism.
  These adjustments ensure the data and narrative are aligned.

**Thirdly,** while H&E staining images have been provided, it would strengthen the histological analysis if Oil Red O staining images were also included, if available.

**Response:**

 We thank the reviewer for this suggestion. Although Oil Red O staining was initiated, the image analysis and quantification were not complete at the time of this revision. As a result, we have **removed all references to Oil Red O staining** from the manuscript to avoid presenting incomplete data. We plan to include these findings in a future update or follow-up publication once the analysis is finalised.

 Finally, the methodology section mentions that "PCR products were then separated on a 1% agarose gel, visualized, and photographed under ultraviolet light," but this data appears to be missing from both the manuscript and the supplementary materials. Please ensure that this data is either included or the statement is revised accordingly.

**Response:**

 We appreciate the reviewer’s attention to this detail. As gel images were not prepared for inclusion in the current version, we have removed this statement to avoid confusion.

 Thank you again for your constructive feedback, which has significantly improved the clarity and quality of our manuscript.

 Best Regards

 Hayder Al-Aubaidy

 On behalf of all authors for this manuscript

# Reviewer report: Reviewer response for version 2

**DOI:** 10.5256/f1000research.156125.r358416

**Published:** 2025-02-05

**Authors:** Xin-Fang Leong

**Affiliations:** 1 Universiti Kebangsaan Malaysia, Bangi, Selangor, Malaysia

**Introduction:**

 1. The introduction mentions that "previous studies have illustrated the action of inflammation and oxidative damage in atherogenic plaques progression," but it does not specify any specific studies or key findings that have directly contributed to this knowledge. 

 2. The introduction could better highlight how the current study fills a gap in the existing literature. What makes pterostilbene and sitagliptin particularly interesting for atherosclerosis? How does this research differ from prior studies on these compounds?

 3. The writing sometimes shifts abruptly between concepts (e.g., from the role of mTOR in autophagy to the effects of pterostilbene). To improve coherence, consider dividing the introduction into clearly defined subsections, such as "Atherosclerosis and Inflammation," "Role of Autophagy in Atherosclerosis," and "Potential Therapeutic Agents," etc.

**Materials & Methods:**

 1. Provide more details on the composition of the atherogenic diet -The diet was a traditional chow with 2% cholesterol? 

 2. Expand on the rationale for sample size and the method of calculation for statistical power. Why 26 male and 54 female rabbits? 

 3. Justify the chosen single dosage for PT and ST and the feeding duration for 8 weeks.

 4. Detail how autophagy and RT-PCR measurements will be quantified and analyzed.

 a) RT-PCR protocol is described, the specific genes of interest (e.g., PI3K, AKT, AMPK, mTOR) are listed but without any context about their relevance to atherosclerosis.  Information about housekeeping genes used for normalization should be included.

 b) The use of LC3B as a marker of autophagy is appropriate, but a brief explanation of how this marker will be measured and explaining why LC3B was chosen as a representative marker would enhance the depth of rationale for the study. 

 5.  Provide more information on the histopathological examination, including scoring criteria and staining methods (the scoring criteria should not be placed in the Results section).

 6. Kruskal-Wallis or Mann-Whitney for histopathological grading? Any comparison done between ST and PT? 

**Results:**

 1. To enhance clarity and avoid redundancy, do refrain from repeating the same data in the text, table, and figure within the Results section. Each element should serve a distinct purpose: the text can summarize key findings, while tables and figures provide visual or detailed data, ensuring they complement each other without overlap. For example: level of F2-isoprostane (data) can be found in body text as well as in the Table and Figure. Same goes to the results for lipid profile. For LC3B - Figure 3 can be removed to avoid redundancy. 

 2. Histopathological examination: The progression of the lesion should be demonstrated by the AC group, while the improvement of the lesion is more effectively represented by the PT or ST groups.

**Discussion:**

 1. Refine interpretations of molecular mechanisms: Emphasize that some of the proposed mechanisms are hypotheses based on indirect evidence and suggest future experimental validation.

 2. Provide more detailed analysis of the clinical relevance of the lipid profile changes, including comparison to existing treatments. 

 3. The role of autophagy in atherosclerosis is discussed, but the potential dangers of excessive autophagy are not considered. In some contexts, autophagy may contribute to cell death, and an overactive autophagic response could potentially exacerbate tissue damage. 

 4. More thoroughly explain how sitagliptin activates AMPK and its role in atherosclerosis beyond glucose regulation.

 5. Address study limitations in more detail.

 6. Specific future research suggestions: Outline clear next steps for validating and translating these findings into clinical practice (e.g. how could pterostilbene or sitagliptin be optimized for clinical use).

**Conclusion:**

 1. The conclusion could be more cautious in attributing the observed effects solely to the PI3K/Akt/mTOR and AMPK/mTOR pathways. Incorporating phrases like "potentially through" or "likely due to" would make the conclusion more nuanced and scientifically precise.

 2. Acknowledging the study’s limitations in the conclusion would be beneficial. For example, noting that the results are based on animal models and that further studies, particularly human clinical trials, are necessary would provide a more balanced perspective.

 3. The conclusion does not address the potential implications of these findings for human clinical practice.

 4. Considering the positive effects of both compounds, the conclusion could briefly explore the potential for combination therapy, highlighting its possible therapeutic advantages.

Is the work clearly and accurately presented and does it cite the current literature?

Yes

If applicable, is the statistical analysis and its interpretation appropriate?

Yes

Are all the source data underlying the results available to ensure full reproducibility?

Yes

Is the study design appropriate and is the work technically sound?

Yes

Are the conclusions drawn adequately supported by the results?

Partly

Are sufficient details of methods and analysis provided to allow replication by others?

Partly

Reviewer Expertise:

Pharmacology, cardiovascular system, animal study

I confirm that I have read this submission and believe that I have an appropriate level of expertise to confirm that it is of an acceptable scientific standard, however I have significant reservations, as outlined above.

# Response

**Published:** 2025-02-07

**Authors:** Hayder Al-Aubaidy

**Affiliations:** La Trobe University, Australia

Dear Dr. Xin-Fang Leong (Reviewer #2):

 Thank you very much for your highly valuable input. 

 Please see below our response to your comments, highlighting the main changes we implemented in the manuscript, version 3:

**Introduction:**

**1. The introduction mentions that "previous studies have illustrated the action of inflammation and oxidative damage in atherogenic plaques progression," but it does not specify any specific studies or key findings that have directly contributed to this knowledge.**

 Thank you for pointing this out. We have revised the introduction to specify key studies that have directly contributed to the understanding of inflammation and oxidative damage in atherogenic plaque progression. For example, Libby et al. (2002) highlighted the role of IL-6 and TNF-α in promoting plaque instability, while Witold et al. (2017) demonstrated the involvement of oxidative stress in endothelial dysfunction and foam cell formation.

**2. The introduction could better highlight how the current study fills a gap in the existing literature. What makes pterostilbene and sitagliptin particularly interesting for atherosclerosis? How does this research differ from prior studies on these compounds?**

 We appreciate this suggestion. The revised introduction now explicitly outlines the gap in existing literature, emphasising the limited data on the combined effects of pterostilbene and sitagliptin on atherosclerosis. We have elaborated on their potential synergistic effects, which differ from prior studies focusing solely on their individual impacts.

**3. The writing sometimes shifts abruptly between concepts (e.g., from the role of mTOR in autophagy to the effects of pterostilbene). To improve coherence, consider dividing the introduction into clearly defined subsections, such as "Atherosclerosis and Inflammation," "Role of Autophagy in Atherosclerosis," and "Potential Therapeutic Agents," etc.**

 To enhance clarity, we have reorganised the introduction into clearly defined subsections: "Atherosclerosis and Inflammation," "Role of Autophagy in Atherosclerosis," and "Potential Therapeutic Agents." This restructuring ensures a smoother transition between concepts.

**Materials & Methods:**

**1. Provide more details on the composition of the atherogenic diet -The diet was a traditional chow with 2% cholesterol?**

 The manuscript now specifies that the atherogenic diet consisted of traditional chow supplemented with 2% cholesterol, following established protocols for inducing atherosclerosis in animal models.

**2. Expand on the rationale for sample size and the method of calculation for statistical power. Why 26 male and 54 female rabbits?**

 We have provided details on the power analysis conducted to determine the sample size, which offers over 85% power to detect significant differences. The unequal male-to-female ratio was due to availability constraints while ensuring both sexes were represented.

**3. Justify the chosen single dosage for PT and ST and the feeding duration for 8 weeks.**

 The dosages for pterostilbene (10 mg/kg) and sitagliptin (12 mg/kg) were based on prior studies demonstrating their efficacy without adverse effects. The eight-week duration aligns with protocols sufficient for the development of atherosclerotic changes and therapeutic assessment.

**4. Detail how autophagy and RT-PCR measurements will be quantified and analyzed.**

**a) RT-PCR protocol is described, the specific genes of interest (e.g., PI3K, AKT, AMPK, mTOR) are listed but without any context about their relevance to atherosclerosis.  Information about housekeeping genes used for normalization should be included.**

 We have elaborated on the relevance of PI3K, AKT, AMPK, and mTOR to atherosclerosis and included GAPDH as the housekeeping gene for normalisation.

**b) The use of LC3B as a marker of autophagy is appropriate, but a brief explanation of how this marker will be measured and explaining why LC3B was chosen as a representative marker would enhance the depth of rationale for the study.** 

 The rationale for selecting LC3B as a marker of autophagy is now explained in detail, highlighting its role in autophagosome formation and relevance in vascular studies.

**5.  Provide more information on the histopathological examination, including scoring criteria and staining methods (the scoring criteria should not be placed in the Results section).**

 We have included comprehensive information on the scoring criteria and staining methods, which were previously only partially described.

**6. Kruskal-Wallis or Mann-Whitney for histopathological grading? Any comparison done between ST and PT?**

 The Kruskal-Wallis test was used for overall comparisons, with Mann-Whitney U tests for pairwise comparisons, including between the PT and ST groups.

**Results:**

**1. To enhance clarity and avoid redundancy, do refrain from repeating the same data in the text, table, and figure within the Results section. Each element should serve a distinct purpose: the text can summarize key findings, while tables and figures provide visual or detailed data, ensuring they complement each other without overlap. For example: level of F2-isoprostane (data) can be found in body text as well as in the Table and Figure. Same goes to the results for lipid profile. For LC3B - Figure 3 can be removed to avoid redundancy.**

 We have streamlined the Results section to avoid redundancy, summarising key findings in the text while using tables and figures to present detailed data.  

**2. Histopathological examination: The progression of the lesion should be demonstrated by the AC group, while the improvement of the lesion is more effectively represented by the PT or ST groups.**

 The revised section now better illustrates lesion progression in the AC group and improvements in the PT and ST groups, with clear histological descriptions supported by representative images.

**Discussion:**

**1. Refine interpretations of molecular mechanisms: Emphasize that some of the proposed mechanisms are hypotheses based on indirect evidence and suggest future experimental validation.**

 We have clarified that the proposed mechanisms are hypotheses based on indirect evidence, suggesting future studies for experimental validation.

**2. Provide more detailed analysis of the clinical relevance of the lipid profile changes, including comparison to existing treatments.**

 The discussion now includes a comparison to existing treatments, highlighting potential complementary roles for pterostilbene and sitagliptin.

**3. The role of autophagy in atherosclerosis is discussed, but the potential dangers of excessive autophagy are not considered. In some contexts, autophagy may contribute to cell death, and an overactive autophagic response could potentially exacerbate tissue damage.**

 We have acknowledged the potential risks of excessive autophagy, which could lead to cell death and exacerbate atherosclerosis in certain contexts.

**4. More thoroughly explain how sitagliptin activates AMPK and its role in atherosclerosis beyond glucose regulation.**

 The revised discussion provides a more thorough explanation of how sitagliptin activates AMPK and its broader role in vascular health beyond glucose regulation.

**5. Address study limitations in more detail.**

 Additional limitations are discussed, including the reliance on animal models and potential sensitivity issues with histological methods.

**6. Specific future research suggestions: Outline clear next steps for validating and translating these findings into clinical practice (e.g. how could pterostilbene or sitagliptin be optimized for clinical use).**

 We have outlined specific next steps for clinical translation, such as optimising dosing, exploring combination therapies, and conducting human trials.

**Conclusion:**

**1. The conclusion could be more cautious in attributing the observed effects solely to the PI3K/Akt/mTOR and AMPK/mTOR pathways. Incorporating phrases like "potentially through" or "likely due to" would make the conclusion more nuanced and scientifically precise.**

 The conclusion now uses terms like "potentially" and "likely" to present a more nuanced interpretation of the mechanisms involved.

**2. Acknowledging the study’s limitations in the conclusion would be beneficial. For example, noting that the results are based on animal models and that further studies, particularly human clinical trials, are necessary would provide a more balanced perspective.**

 We have explicitly noted that the findings are based on animal models and highlighted the need for human clinical trials.

**3. The conclusion does not address the potential implications of these findings for human clinical practice.**

 The potential implications for human atherosclerosis management are discussed, considering both agents as adjunctive therapies.

**4. Considering the positive effects of both compounds, the conclusion could briefly explore the potential for combination therapy, highlighting its possible therapeutic advantages.**

 We have added a brief discussion on the potential benefits of combination therapy, considering the complementary mechanisms of pterostilbene and sitagliptin.

 Thank you again for your constructive feedback, which has significantly improved the clarity and quality of our manuscript.

 Best Regards,

 Hayder Al-Aubaidy

 On behalf of all authors for this manuscript

# Reviewer report: Reviewer response for version 2

**DOI:** 10.5256/f1000research.156125.r208141

**Published:** 2023-09-22

**Authors:** Jelica Grujic Milanovic

**Affiliations:** 1 University of Belgrade, Belgrade, Serbia

Authors well addressed my previous comments. The paper improved very much.

Is the work clearly and accurately presented and does it cite the current literature?

Yes

If applicable, is the statistical analysis and its interpretation appropriate?

Yes

Are all the source data underlying the results available to ensure full reproducibility?

Yes

Is the study design appropriate and is the work technically sound?

Yes

Are the conclusions drawn adequately supported by the results?

Yes

Are sufficient details of methods and analysis provided to allow replication by others?

Yes

Reviewer Expertise:

Oxidative stress, polyphenols, hypertension, cardiovascular diseases

I confirm that I have read this submission and believe that I have an appropriate level of expertise to confirm that it is of an acceptable scientific standard.

# Reviewer report: Reviewer response for version 1

**DOI:** 10.5256/f1000research.143454.r199143

**Published:** 2023-09-14

**Authors:** Jelica Grujic Milanovic

**Affiliations:** 1 University of Belgrade, Belgrade, Serbia

The article by Sahib *et al.* has been clearly defined. The methodology applied is overall correct. The authors support their paper with recent references.

 However, the following changes to the main text are suggested:

- In the results, use the abbreviation TC instead of total cholesterol.
- In Figure 1 and Table 1 the authors presented the same results. The authors must improve this part of the results.
- In Figure 1, use the same colors for marking as a group as in Figure 1/4/5.
- Does the letter F in Figure 3 stand for the ST group?

Is the work clearly and accurately presented and does it cite the current literature?

Yes

If applicable, is the statistical analysis and its interpretation appropriate?

Yes

Are all the source data underlying the results available to ensure full reproducibility?

Yes

Is the study design appropriate and is the work technically sound?

Yes

Are the conclusions drawn adequately supported by the results?

Yes

Are sufficient details of methods and analysis provided to allow replication by others?

Yes

Reviewer Expertise:

oxidative stress, polyphenols, hypertension, cardiovascular diseases

I confirm that I have read this submission and believe that I have an appropriate level of expertise to confirm that it is of an acceptable scientific standard, however I have significant reservations, as outlined above.

# Response

**Published:** 2023-09-15

**Authors:** Hayder Al-Aubaidy

**Affiliations:** La Trobe University, Australia

Dear Professor Grujic,

 Many thanks for reviewing the manuscript and providing us with your comments. 

 We confirm addressing all your comments in Version 2 of the manuscript. A summary of the changes has been listed below:

 The article by Sahib et al. has been clearly defined. The methodology applied is overall correct. The authors support their paper with recent references.

 However, the following changes to the main text are suggested:

 In the results, use the abbreviation TC instead of total cholesterol.

**Response:** Thank you for your comment. This has been fixed across the results section.

 In Figure 1 and Table 1 the authors presented the same results. The authors must improve this part of the results.

**Response:** We have removed Figure 1 (serum lipid parameters) and kept Table 1 to avoid duplication of data. We have renamed the remaining figures accordingly.

 In Figure 1, use the same colors for marking as a group as in Figure 1/4/5.

**Response:** Figure 1 – Serum lipid parameters has been removed from the manuscript as per the comment above. 

 Does the letter F in Figure 3 stand for the ST group?

**Response:** In this figure, letter F shows complicated atherosclerotic changes - hemorrhagic thrombus as seen in the atherogenic study cohort (no supplementations). The changes in the sitagliptin group are shown in letter B. Please refer to the figure legend for more details about the changes in the other study groups.

 Best Regards

 Prof. Hayder Al-Aubaidy

 On behalf of all authors for this manuscript
